# Single nucleotide variants in *UNC13C* associated with neurodevelopmental disorders affect ethanol sensitivity in *Drosophila*

**DOI:** 10.1016/j.bbrep.2025.102375

**Published:** 2025-11-28

**Authors:** Franz Müller, Sonja Neuser, Gaurav Shrestha, Netra P. Neupane, Katharina J. Götze, Nicola Brunetti-Pierri, Gaetano Terrone, Alexandre Reymond, Koen L. van Gassen, Eva Brilstra, Katharina Steindl, Anais Begemann, Anita Rauch, Jonathan Rips, Duha Fahham, Tahsin Stefan Barakat, Olivier Patat, Jérémie Mortreux, Matthew Hoi Kin Chau, Jill A. Rosenfeld, Elizabeth Mizerik, Swati Srivastava, Xi Luo, Anne-Kristin Dahse, Nicole Scholz, Joydip Das, Gregg Roman, Tobias Langenhan, Rami Abou Jamra, Achmed Mrestani, Dmitrij Ljaschenko

**Affiliations:** a Rudolf Schönheimer Institute of Biochemistry, Division of General Biochemistry, Medical Faculty, Leipzig University, Leipzig, 04103, Germany; b Institute of Human Genetics, University of Leipzig Medical Center, Leipzig, 04103, Germany; c Department of Biology, University of Mississippi, Oxford, MS, 38677, USA; d Department of BioMolecular Sciences, University of Mississippi, Oxford, MS, 38677, USA; e Department of Pharmacological and Pharmaceutical Sciences, College of Pharmacy, University of Houston, Houston, TX, 77204-5037, USA; f Department of Translational Medicine, Federico II University, Naples, 80131, Italy; g Telethon Institute of Genetics and Medicine, Pozzuoli, 80078, Italy; h Genomics and Experimental Medicine Program, Scuola Superiore Meridionale (SSM, School of Advanced Studies), Naples, 80134, Italy; i Center for Integrative Genomics, University of Lausanne, Lausanne, 1015, Switzerland; j Department of Genetics, University Medical Center Utrecht, Utrecht, 3584 CX, the Netherlands; k Institute of Medical Genetics, University of Zürich, Schlieren-Zürich, 8952, Switzerland; l Genetic Department, Hadassah Hebrew University Hospital, Jerusalem, 91120, Israel; m Department of Clinical Genetics, Erasmus MC University Medical Center, Rotterdam, 3015 CN, the Netherlands; n Service de génétique médicale, Hôpital Purpan, CHU Toulouse, Toulouse, 31300, France; o Laboratoire de Biologie Médicale Multi-Sites AURAGEN, Lyon, 69003, France; p Department of Obstetrics and Gynaecology, The Chinese University of Hong Kong, 999077, Hong Kong, China; q The Chinese University of Hong Kong-Baylor College of Medicine Joint Center for Medical Genetics, 999077, Hong Kong, China; r Hong Kong Hub of Paediatric Excellence, The Chinese University of Hong Kong, 999077, Hong Kong, China; s Department of Molecular and Human Genetics, Baylor College of Medicine, Houston, TX77030, USA; t Texas Children's Hospital, Houston, TX, 77030, USA; u Baylor Genetics, Houston, TX, 77021, USA; v Comprehensive Cancer Center Central Germany, Leipzig University, Leipzig, 04103, Germany; w Institute of Biology, Faculty of Life Sciences, Leipzig University, Leipzig, 04103, Germany; x Department of Neurology, Leipzig University Medical Center, Leipzig, 04103, Germany

**Keywords:** Unc13, UNC13C, Dunc13, Chemical synapse, Neurodevelopmental disease, Ethanol sensitivity, Molecular dynamics simulation

## Abstract

UNC13s are presynaptic proteins essential for neurotransmitter release at chemical synapses. In this study, we present eleven patients from nine families with severe neurodevelopmental impairments, who carry rare, biallelic *UNC13C* single-nucleotide variants (SNVs). Six missense variants, each identified in compound heterozygosity in one of three of these patients, were introduced into the *Drosophila melanogaster* ortholog *unc13* using a previously established CRISPR/Cas9-based method for rapid and scarless genomic modifications, hypothesising that they underlie the observed clinical manifestations. However, none of the introduced mutations influenced Mendelian ratios, negative geotaxis, or lifespan of the fruit flies. Interestingly, two variants located outside the gene regions encoding known UNC13C domains caused a decreased ethanol sensitivity in *Drosophila*, while the Thr1729Met substitution within the C_1_ domain resulted in increased ethanol sensitivity. Molecular dynamics simulations of the latter mutant gene product suggested that the altered protein conformation enhances exposure of the ethanol-binding site, thereby increasing sensitivity to ethanol. These findings reinforce previous evidence highlighting the critical role of the C_1_ domain in ethanol sensitivity. Given the involvement of the C_1_ domain in synaptic plasticity this result might implicate an influence of the Thr1729Met on synaptic function.

## Highlights

• **Eleven patients with neurodevelopmental disorders carry rare *UNC13C* variants**.
• **Six *UNC13C* mutations modelled in *Drosophila* via scarless CRISPR/Cas9 genome editing**.
• **Two *UNC13C* variants increase ethanol tolerance in *Drosophila***.
• **Thr1729Met impairs ethanol tolerance by altering *UNC13C* membrane interaction**.

## 1 Introduction

UNC13 proteins are key molecules in molecular processes that underlie chemical synaptic transmission [[1], [2], [3]]. Transcripts and proteins of three *UNC13* genes (*UNC13A*, *B*, and *C* in mammalian organisms, also known as *Munc13-1*, *-2*, and *-3*) play a role in the mammalian nervous systems [2]. The homologs in *Caenorhabditis elegans* and *Drosophila melanogaster* are referred to as *unc13* (in *Drosophila* also as *dunc-13*). Both animals possess only one *unc13* gene [[1], [2], [3]]. UNC13 proteins in mammals and invertebrates share homologous domains and play a central role in vesicle release [2,4].

**Fig. 1:**
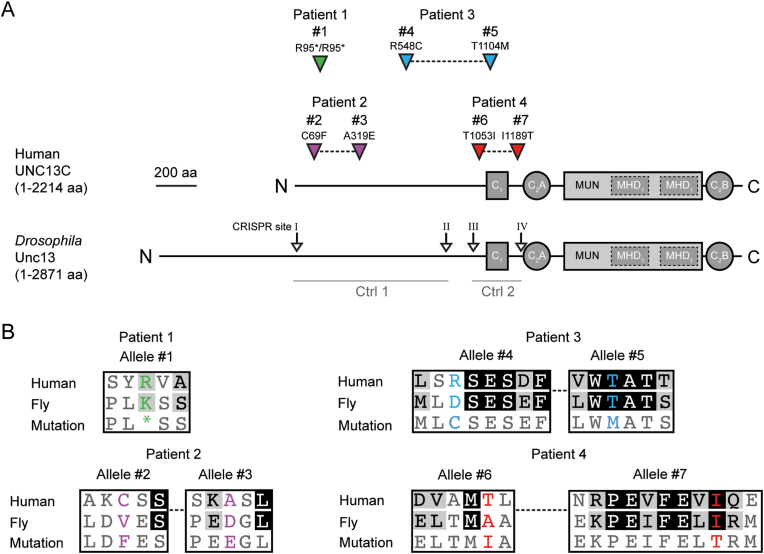
**Reconstruction of neurodevelopmental disorder-associated *UNC13C* SNVs in *Drosophila*. (A)** Variant positions in the UNC13C protein (isoform: UniProt accession number Q8NB66), aligned to the *Drosophila* Unc13 protein (isoform UNC-13A) of patients 1–4. Patient 1, homozygous variant #1 in green; patient 2: variant #2/#3 in magenta; patient 3: variant #4/#5 in blue; patient 4: variant #6/#7 in red. Known domains of UNC13C and *Drosophila* Unc13 are shown as grey boxes or circles. CRISPR/Cas9 *gRNA* target site pairs I/II and III/IV used for mutagenesis are shown. Homology-directed repair (HDR) plasmids with sequences including silent mutations (Ctrl 1, Ctrl 2) were used to create negative control animals. Amino acid numbering refers to the human protein. **(B)** Alignment of the amino acid sequence of human UNC13C and *Drosophila* Unc13 in the vicinity of the variants shown in (A). Mutations are colour-coded as in (A). Upper line, sequence in the consensus human genome; middle line, sequence in *Drosophila*; bottom line, variant found in patients and introduced as a mutation into *Drosophila*. Residues highlighted with a black background are conserved between the species. Residues, highlighted with a grey background, show structurally similar amino acids. Adapted from Ref. [6].

The C-terminal region of UNC13 contains the C_1_, two C_2_ (C_2B_ and C_2C_), and the MUN domains [2,5](Fig. 1A). The MUN domain converts plasma membrane-bound syntaxin from a closed to an open conformation, which engages with other proteins of the SNARE complex, resulting in Ca^++^-dependent vesicle fusion and the release of vesicle content into the synaptic cleft [[7], [8], [9], [10]]. The C_1_ and C_2_ domains cooperate in the inhibition of the MUN domain [11], which is lifted when C_1_ binds diacylglycerol (DAG) or when the C_2B_ domain binds Ca^++^ or phospholipids [12]. The N-terminus of UNC13 is assumed to coordinate the stable positioning of the protein at vesicle release sites [13,14].

In humans, specific variants of the genes *UNC13A* and *UNC13B* are associated with the severe degenerative motoneuron disease amyotrophic lateral sclerosis (ALS), as well as with bipolar affective disorder [[15], [16], [17]]. Further, mRNA of *UNC13A* is misprocessed in individuals with ALS [18,19]. Interestingly, the effectiveness of ALS treatment with lithium varies between patients with different *UNC13A* variants [20]. In addition, biallelic loss of function of UNC13A has been associated with congenital encephalopathy and a severe neuromuscular phenotype [21].

In this study, we report eleven children with rare single nucleotide variants in *UNC13C* (NM_001080534.3) who suffer from different ailments, including global developmental delay, microcephaly, autism spectrum disorder, and various brain malformations. Four patients from 2 families (1 and 9–11) carry a homozygous nonsense variant, which probably results in non-sense mediated decay of mRNA or an early truncation of the amino acid sequence rendering the gene product devoid of all the functionally characterised protein domains. Each patient 2, 3, and 4 carries compound heterozygous (biallelic) single nucleotide missense variants, one inherited from each of their clinically unaffected parents. No other disease-explaining variants, which could account for the phenotypes, were found in patients 2, 3 and 4. According to international variant classification guidelines [22], all variants are accounted as variants of unknown significance. We hypothesised that these variants cause the observed clinical symptoms. To test this hypothesis, the *UNC13C* variants were introduced into the homologous positions of the *unc13* gene of the genetically and experimentally highly accessible model organism *Drosophila,* and the resulting phenotypes were functionally assessed. Rare variants identified in a biallelic state in additional patients, which we discovered later, are presented but not analysed in this study.

## 2 Materials and methods

### 2.1 Recruitment

This study was approved by the Leipzig University (Germany) ethics committee (402/16-ek) and carried out in accordance with the Declaration of Helsinki of the World Medical Association. Written consent of the legal guardians of the presented individuals regarding publishing genetic and clinical data was obtained, or limited data are presented under a retrospective review protocol approved by the Baylor College of Medicine IRB. Privacy rights of human subjects were respected throughout the study. The cohort was assembled by the Institute of Human Genetics in Leipzig and via matchmaking using GeneMatcher [23] from institutions in Italy, Switzerland, France, the Netherlands Israel, and USA.

### 2.2 Molecular reagents

Sequences of primers used throughout this study are listed in Table S1.

### 2.3 sgRNA plasmids

*sgRNA* plasmids to guide the Cas9 enzyme to the desired genomic position were made in a previous study [6]. There, CRISPR/Cas9 target sites (i.e., CRISPR sites I-IV, Fig. 1A) located within the *unc13* locus were identified using the "CRISPR Optimal Target Finder" [24]. Genomic sequences were verified by Sanger-sequencing the PCR fragments covering the predicted sites. Target-specific sequences were synthesised as 5′-phosphorylated oligonucleotides, then annealed and ligated into the BbsI sites of the *pU6-BbsI-chiRNA* vector [25]. *sgRNA* plasmids used in this study are listed in Table S2.

### 2.4 *unc13* homology-directed repair vectors

Homology-directed repair (HDR) vectors without *sgRNA* binding site modifications for mutations #2, #3, #4, #5, #6 and #7 (Fig. 1A) were made in a previous study [6], and already include *sgRNA* binding site modifications (see below) for mutations #2 and #3. In short, for mutations #2, #3 and #4, a 4.3 kb fragment was PCR-amplified from *w1118* flies using primers am_226F/am_223R. After gel purification, the product was digested with SacII and AvrII and ligated into a 2.8 kb backbone fragment of SacII/AvrII-digested *pHD-DsRed-attP* (pTL620, Addgene #51019), resulting in pAM66. For mutations #5, #6 and #7 (Fig. 1A), a 3.9 kb fragment was PCR-amplified from *w1118* genomic DNA using primers am_227F and am_225R, then gel-purified, digested with SacII/AvrII and ligated into the same *pHD-DsRed-attP* (pTL620) producing pAM67. Point mutations were introduced by QuikChange site-directed mutagenesis using Pfu DNA polymerase (Promega). A DpnI digest was performed to eliminate the bacterial plasmid template. Primers were designed for optimal *Drosophila* codon usage and contained the mutated nucleotides flanked by 12–21 bp long homologous sequences. To prevent unintended Cas9 cleavage after the incorporation of the HDR sequence at the according genomic position in *Drosophila*, silent mutations were introduced into *sgRNA* binding sites and PAM sites of the HDR vectors for mutations #4, #5, #6 and #7, following the earlier procedure for mutations #2, #3 that was described previously [6]. These modifications were performed by GenScript (USA). The vectors, without introduced patient mutations, were also used for embryo injection to create negative control genotypes *unc13*^*Ctrl 1*^ and *unc13*^*Ctrl 2*^ and to test whether the mutagenesis procedure itself created unspecific negative consequences for the gene product. HDR plasmids used in this study are listed in Table S3.

### 2.5 *ovo*^*D*^-assisted *unc13* targeting with CRISPR/Cas9

Fly strains that were generated in this study are listed in Table S4. Additional strains that were used are listed in Table S5. *Drosophila* embryo injections were performed at BestGene (USA). To generate *unc13* alleles, *ovo*^*D*^ co-selection was employed, as previously demonstrated [26]. All crossing and selection procedures are described in Fig. S3. Male flies carrying the dominant-negative *ovo*^*D1*^ mutation on the X chromosome (BDSC #1309) were crossed with virgin females expressing *nos-Cas9* (BDSC #78782). Embryos from this cross were injected with pAM63 (Addgene plasmid #111142, *pCFD3-ovo*^*D1*^*-2*) along with the 5' and 3' sgRNA target plasmids and one of the HDR donor plasmids [26]. In flies where Cas9 cutting occurred, the pAM63-encoded *sgRNA* would silence the dominant-negative *ovo*^*D1*^ allele and render the offspring female flies fertile. In most cases where this happened, the introduction of the *unc13* HDR sequence was also successful, rendering the mutagenesis procedure very efficient. The resulting females were crossed with males, which carried *RFP/RFP* on the 4th chromosome. The offspring males were single-crossed with females carrying suitable phenotypic markers on the 4th chromosome (Table S5) and genotyped (PCR, Sanger sequencing) for the presence of the desired mutation with appropriate primers. The resulting flies had the genotype *unc13*^*X*^*/ci*^*D*^ (Table S4), where *X* stand for mutations #2 - #7, or *unc13*^*Ctrl 1*^, *unc13*^*Ctrl 2*^.

### 2.6 Genotyping of mutant fly strains

Genotyping was performed via Sanger sequencing of PCR fragments to select fly strains with the desired mutation. Additionally, to exclude unwanted off-target mutations by secondary Cas9 cuts, the CRISPR/Cas9 target sites (i.e., *sgRNA* binding) were also analysed. Primers used to test for the presence of mutations and to exclude that the CRISPR/Cas9 target sites were unintentionally mutated after targeting are listed in Table S1. The same primers were used to test for the successful mock mutagenesis to create the negative control genotypes *unc13*^*Ctrl 1*^ and *unc13*^*Ctrl 2*^ (Fig. 1A). When silent modifications of *sgRNA* binding sites and PAM to prevent repeated Cas9 cutting were present in the genome, the mutageneses to create *unc13*^*Ctrl 1*^ and *unc13*^*Ctrl 2*^ were deemed successful.

### 2.7 Sanger sequencing

Genomic DNA was extracted using Macherey-Nagel's NucleoSpin tissue kit. DNA fragments were amplified using PCR with suitable primers. Gel electrophoresis and the QIAquick gel extraction kit (QIAGEN) were used to purify DNA. Water, forward and reverse primers, and DNA were mixed in a 1.5 ml tube and sent to Microsynth for sequencing. The results were analysed with the ApE plasmid editor.

### 2.8 *Drosophila* husbandry

Experimental fly crosses were maintained at 25 °C on standard cornmeal food (4.5 g agar, 20 g beet syrup, 72.2 g malt extract, 9 g soy flour, 16.3 g yeast, 72.2 g corn flour, 1.45 g methyl 4-hydroxybenzoate, 5.7 g propionic acid in 1 L of water) on a 12-h light/dark cycle.

### 2.9 Mendelian ratio experiments

**Fig. 2:**
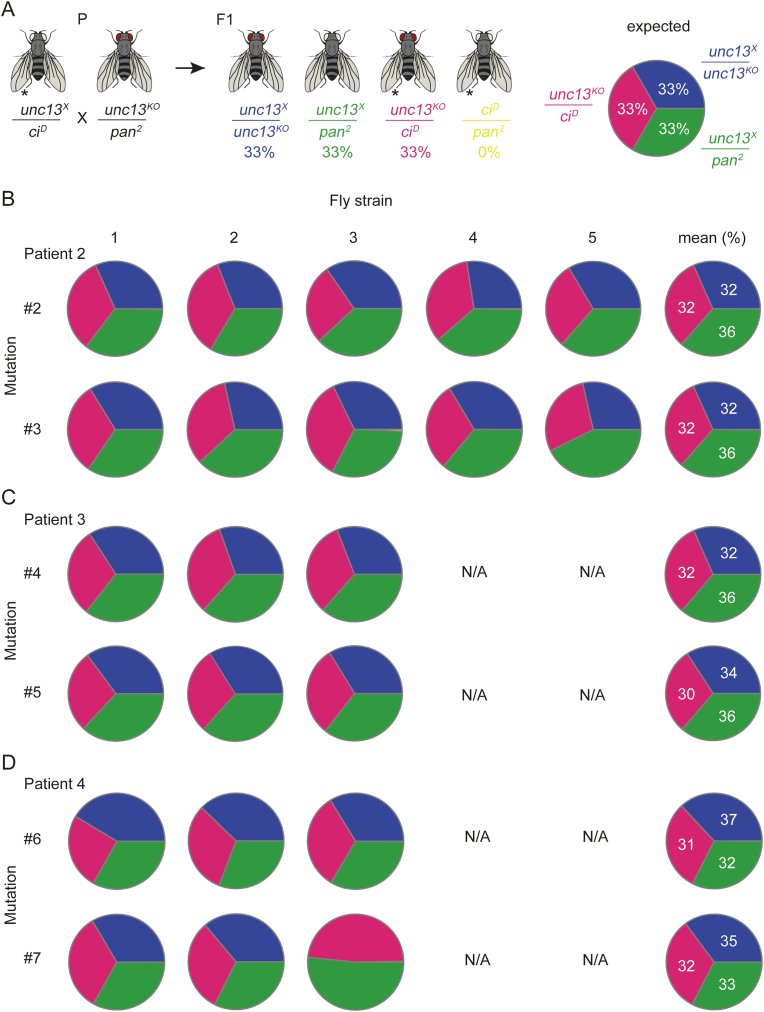
Mendelian experiments show no clear effect of point mutations. **(A)** Crossing scheme and expected Mendelian ratio to test the influence of *Drosophila unc13* point mutations (*unc13*^*X*^) when crossed over *unc13*^*KO*^ (blue). P, parental generation; F1, first filial generation. All genotypes of F1 can be determined using dominant phenotypic markers (red vs white eyes and regular vs shortened wing vein marked with ∗). The *ci*^*D*^*/pan*^*2*^ combination (yellow) is lethal; no flies should hatch. 33.3 % of the three other genotypes are expected, if the mutation has no effect. *unc13*^*KO*^/*ci*^*D*^, magenta; *unc13*^*X*^*/unc13*^*KO*^, blue; *unc13*^*X*^/*pan*^*2*^, green. **(B, C, D, E)** Mendelian ratios for mutations of patient 2 (mutation #2 and #3), patient 3 (mutation #4 and #5) and patient 4 (mutation #6 and #7) genotypes, respectively. Patient 2: five fly strains were examined. Patients 3 and 4: three fly strains were examined per genotype. Mean shows average results from all fly strains in one genotype. Fly strain 3 of mutation #7 was excluded from calculating the average. Number of counted flies for each mutation, not including strain 3 of mutation #7: #2, 1579; #3, 1584; #4, 1103; #5, 977; #6, 1073; #7, 717. Due to rounding, percentages do not always add up to 100 %.

**Fig. 3:**
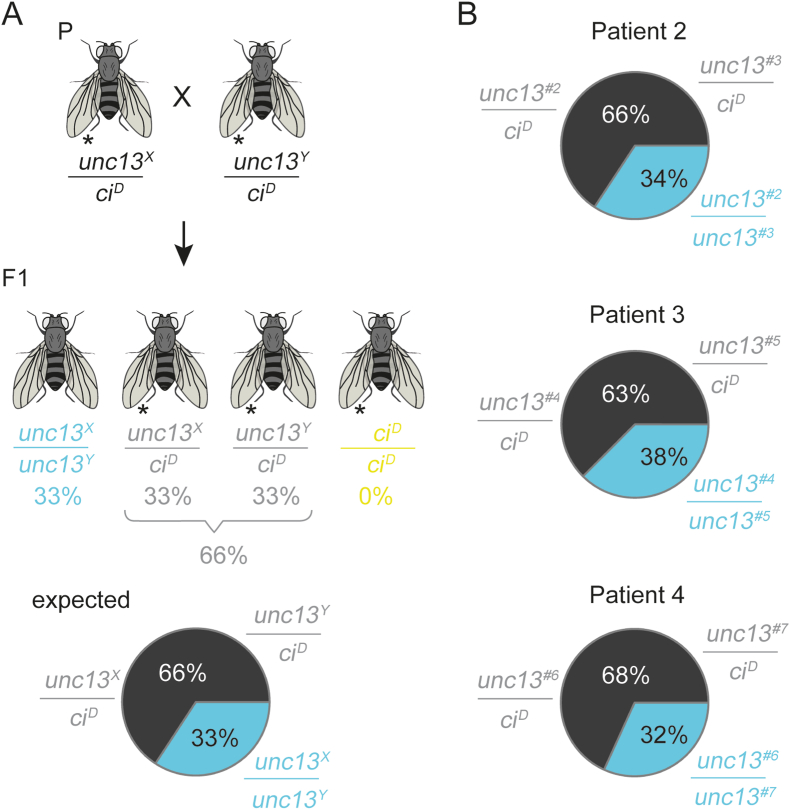
**Mendelian ratios in compound heterozygous crosses simulating the patient genotypes do not show an effect of mutations**. (**A)** Crossing scheme and expected Mendelian ratio to test the influence of compound heterozygous *Drosophila* genotypes that recapitulate the genetic situation in patients (*unc13*^*X*^*/unc13*^*Y*^, turquoise). P, parental; F1, first filial generations. The *ci*^*D*^*/ci*^*D*^ combination (yellow) is lethal and should not occur among hatched F1 flies. If the two mutations combined (*unc13*^*X*^*/unc13*^*Y*^) have no effect, 33.3 % of the flies should be *unc13*^*X*^*/unc13*^*Y*^ (regular wing vein). *unc13*^*X*^*/ci*^*D*^ (33.3 %) and *unc13*^*Y*^*/ci*^*D*^ (33.3 %) cannot be differentiated since both carry the same phenotypic marker (short wing vein, marked with ∗) and amount together to 66.6 % (dark grey). **(B)** Mendelian ratios for mutation *unc13*^*#2*^*/unc13*^*#3*^ (patient 2), *unc13*^*#4*^*/unc13*^*#5*^ (patient 3) and *unc13*^*#6*^*/unc13*^*#7*^ (patient 4), respectively. Strains that were used for the crosses are shown in Table S4. Number of counted flies for each experiment: #2/#3, 359; #4/#5, 264; #6/7, 327. Due to rounding, percentages do not always add up to 100 %.

To assess the impact of the introduced mutations *unc13*^*X*^ on the function of the gene product, when combined with *unc13*^*KO*^, 21 virgin females of the genotype *unc13*^*KO*^*-Act-GFP/pan*^*2*^ (abbreviated as *unc13*^*KO*^*/pan*^*2*^) were crossed with 7 *unc13*^*X*^*/ci*^*D*^ males (Fig. 2A). To test for an effect of the mutations when simulating the compound heterozygous patient genotype *unc13*^*X*^*/unc13*^*Y*^*,* the same number of flies was used to cross *unc13*^*X*^*/ci*^*D*^ with *unc13*^*Y*^*/ci*^*D*^ (Fig. 3A)*.* The parental generation of flies was transferred to a fresh vial every 2–3 days. 21 days after the relocation to a new vial, the adult individuals of the F1 generation were sorted and counted according to phenotypic markers (Figs. 2A and 3A), and the proportion of different phenotypes was determined. Three vials per cross, but not the first vial, were used to determine Mendelian ratios. The results from different fly strains of the same mutation were very similar. Therefore, the results were pooled to give a mean ratio (Fig. 2B). In control experiments (Figs. S1 and S2) the same approach as described above was used.

### 2.10 Negative geotaxis assay

**Fig. 4:**
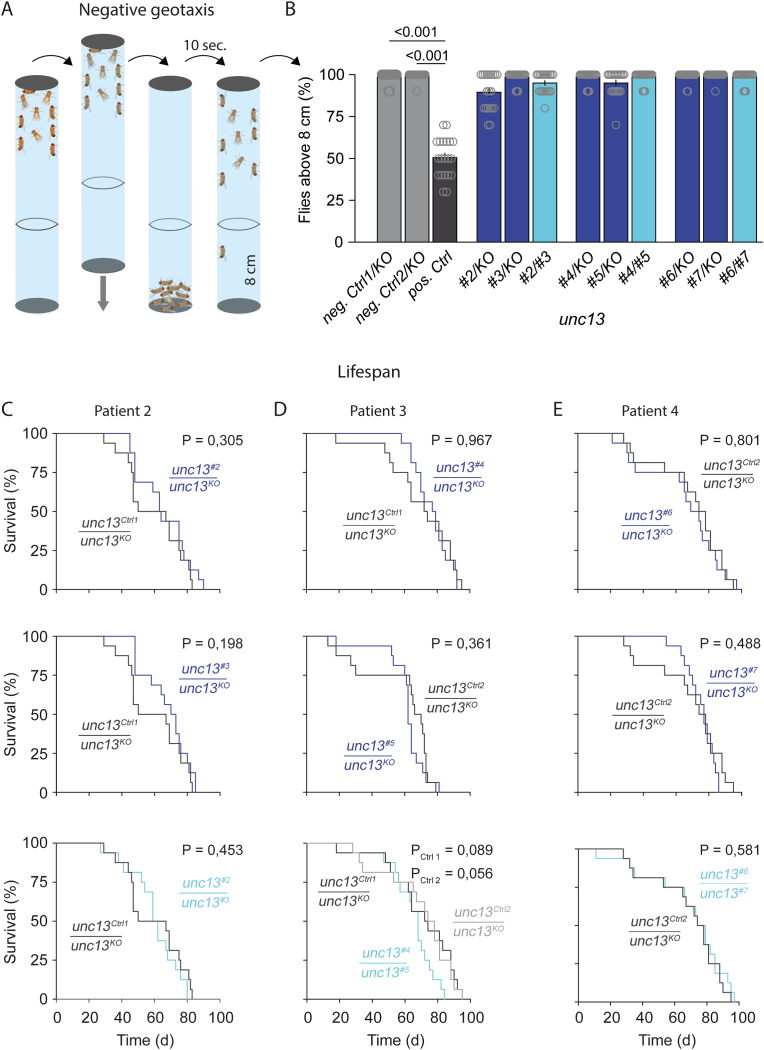
Negative geotaxis and lifespan experiments reveal no clear effect of the mutations. **(A)** Setup for testing negative geotaxis in *Drosophila*. Flies in transparent cylinders were gently knocked to the ground. The number of flies, which climbed higher than 8 cm within 10 s, were counted and presented as percentages in (B). (**B)** Percentage of flies (with SEM) which climbed higher than 8 cm within 10 s in 20 runs (n = 20). *unc13*^*X*^*/unc13*^*KO*^, blue; patient genotypes *unc13*^*X*^*/unc13*^*Y*^, turquoise. Except for the positive control (*Cirl*^*KO*^, dark grey), none of the genotypes showed a significant difference to the respective control genotypes *unc13*^*Ctrl 1*^*/unc13*^*KO*^ and *unc13*^*Ctrl 2*^*/unc13*^*KO*^ (both in light grey). All p-values are shown in Table S7. Since data were non-normally distributed, significance was tested using ANOVA on ranks (Kruskal-Wallis) followed by Dunn's for multiple comparisons. Every group was compared to every other group. Related to Table S6. **(C, D, E)** Lifespan assay results of mutations from patients 2 (C), 3 (D) and 4 (E), when recapitulated in *Drosophila,* are presented as Kaplan-Meier survival curves. *unc13*^*X*^*/unc13*^*KO*^ genotypes are presented in blue. Genotypes, which recreate the compound heterozygous genotypes, i.e., *unc13*^*X*^*/unc13*^*Y*^, are shown in turquoise (lower panels). Statistical comparisons to the respective survival curves of negative control flies were done with Survival LogRank tests. P-values are given in the upper right corner of each panel. Lower panel in D shows p-values from comparisons to both control genotypes since *unc13*^*#4*^ and *unc13*^*#5*^ require different controls. Eight isolated male and eight isolated female flies were used to result in N = 16, which was set as 100 %.

Five male and five female flies with unfolded wings but not older than one day after hatching were anaesthetised with CO_2_ and placed into a 21 cm long transparent cylinder with a diameter of 4.6 cm. After giving flies 1 h for recovery, cylinders containing either flies of interest or control genotypes were secured in a self-made device. This device was knocked to the table five times with a force strong enough for all flies to fall to the ground. Similar forces were applied to all genotypes since all cylinders were fixed in the same device. After being knocked to the ground, flies tend to climb upwards in the cylinder (negative geotaxis, Fig. 4A), which was video recorded. The procedure was repeated nine more times with 1 min breaks between the runs (n = 10). The video recordings were analysed by counting the number of flies which climbed higher than 8 cm after 10 s. This procedure was repeated with another ten freshly hatched flies in a separate vial, with very similar results. Therefore, the two sets of flies of the same genotype were pooled to give n = 20 per genotype.

### 2.11 Lifespan assay

16 freshly hatched flies (eight male and eight female, not older than 24 h) were individually transferred into separate transparent plastic vials. The isolation of flies was implemented to eliminate social influences such as mating or aggressive behaviour. The vials, measuring 6.3 cm in length and 2.5 cm in diameter, were partially filled with a thin layer of food paste (<3 mm thick). The procedure ensured adequate hydration to prevent premature mortality due to desiccation while avoiding the risk of flies drowning in the paste. Flies were transferred to fresh vials twice weekly without using CO_2_ anaesthesia. Their survival status was monitored three times per week. The percentage of surviving flies was plotted against time to generate Kaplan–Meier survival curves. Median survival time was calculated using Sigmaplot (SyStat) software.

### 2.12 Ethanol sensitivity assay

**Fig. 5:**
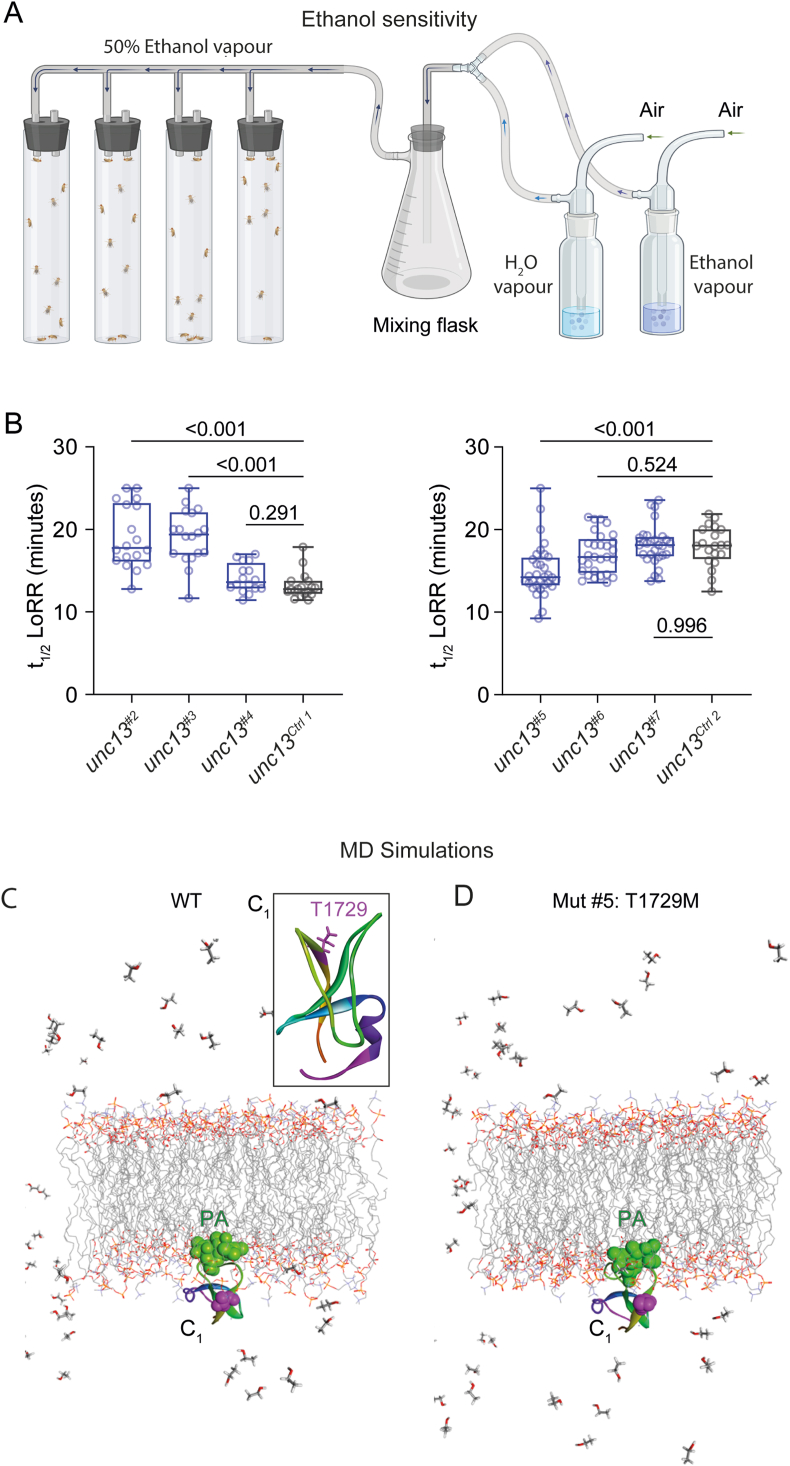
Three mutations show a pronounced distortion in ethanol sensitivity behaviour. (**A)** Illustration of the ethanol sensitivity assay in which flies are exposed to ethanol/water vapour. The time at which half of the flies lose their righting reflex, i.e., fail to stand up after tapping the vial, is registered. (**B)** The times when 50 % of flies lost their righting reflex (LoRR) are plotted as box plots with medians, 25th and 75th percentiles, the minimal and the maximal values, overlaid with the values from individual runs, which are shown as circles. Grey, controls; blue, homozygous *unc13*^*X*^. Data were distributed non-normally. *unc13*^*#2*^*, unc13*^*#3*^*, unc13*^*#4*^ were compared to *unc13*^*Ctrl 1*^ and *unc13*^*#5*^*, unc13*^*#6*^*, unc13*^*#7*^ were compared to *unc13*^*Ctrl 2*^ using the Kruskal-Wallis test followed by Dunn's test for multiple comparisons. unc13^#2^, n = 18; unc13^#3^, n = 18; unc13^#4^, n = 16; unc13^Ctrl 1^, n = 18; unc13^#5^, n = 32; unc13^#6^, n = 29; unc13^#7^, n = 29; unc13^Ctrl 2^, n = 20. Related to Table S8. **(C, D)** Molecular dynamics simulations of the structural consequences of the T1729 M (Amino acid numbering refers to the *Drosophila* isoform A). Inset in (D) shows the structure of the C_1_ domain of wild-type *Drosophila* Unc13 with the ethanol-sensitive residue T1729 (pink, position of mutation #5). The structural model was generated using the rat Munc13-1 C_1_ NMR structure (PDB ID: 1Y8F) as a template in PyMOL. The threonine residue at position 1729 was mutated to methionine, which increases ethanol sensitivity in *Drosophila*. The primary structure of the *Drosophila* Unc13C_1_ (UniProt accession number: Q8IM87) is QHNFLLW**T**ATSPTYCYECEGLLWGIARQGVRCTECGVKCHEKCKDLL NADC. Bold T, the mutated threonine. The structure of the *Drosophila* Unc13-phorbol 13-acetate-membrane-ethanol system is shown in C (WT) and D (mutation #5, T1729 M). The phorbol 13-acetate-bound C_1_ domain was embedded in a 100 % phosphatidylserine solvent with 1 % ethanol. The number of phosphatidylserine molecules in the upper leaflet was 64, and in the lower leaflet, 58, for both systems. Phorbol 13-acetate (PA) and T1729 are represented by green CPK (Corey, Pauling, Koltun space-filling model) and pink CPK, respectively. The lipid tails are depicted as grey wavy lines. Hydrogen atoms are removed from lipids for better visualization. Ethanol molecules are indicated by sticks, while water molecules in the system are not shown.

All mutations and 4th chromosome control genotypes (*unc13*^*Ctrl 1*^ and *unc13*^*Ctrl 2*^) tested in the Loss-of-Righting Reflex assay (LoRR, i.e. ethanol sensitivity assay) were placed in the Roman lab Canton S strain background by replacing the 1st, 2nd, and 3rd chromosomes. Alcohol sedation sensitivity was measured using the LoRR assay [27]. For this assay, 30 male flies for each genotype were collected, placed in food vials, and held at 25 °C for 24 h before the assay. During the experiment, the flies were exposed to 50:50 ethanol:water vapour generated by blowing fresh air through 95 % ethanol and Milli Q water at 500 ml/min (Fig. 5A). The number of sedated flies was counted every 5 min by gently tapping the vial containing flies and determining how many had lost their righting reflex (LoRR). Flies reached the LoRR criteria when they fell on their back or side for over 3 s after tapping the vial. The time for 50 % LoRR was calculated using the forecast function in Microsoft Excel. Thus, higher values on the ordinates correspond to higher mean alcohol resistance (Fig. 5B). The experiment was repeated with different sets of flies. unc13^#2^, n = 18; unc13^#3^, n = 18; unc13^#4^, n = 16; unc13^Ctrl 1^, n = 18; unc13^#5^, n = 32; unc13^#6^, n = 29; unc13^#7^, n = 29; unc13^Ctlr 2^, n = 20.

### 2.13 Molecular docking simulations of mutation #5 T1729 M

#### 2.13.1 Homology modelling

The C_1_ domain of *Drosophila* Unc13 shares ∼92 % sequence similarity with the rat Munc13-1 C_1_ domain. The homology model for *Drosophila* Unc13C_1_ was generated (inset in Fig. 5C) using the rat Munc13-1 C_1_ NMR structure (PDB ID: 1Y8F) as a template in PyMOL 3.0.3. Energy minimisation was performed using the Molecular Operating Environment (MOE) 2023, applying the Amber 10 force field with a 0.1 RMS kcal/mol/Å^2^ gradient. The structure of the C_1_ domain carrying mutation #5, T1729 M, was generated following the same procedure.

#### 2.13.2 Molecular docking

Phorbol esters mimic the endogenous Unc13 activator diacylglycerol (DAG) and are frequently used for Unc13 studies. Unc13 binds DAG/phorbol ester at the C1 domain and associates itself with the plasma membrane in its activation process. To model this, we built a protein-ligand-membrane system [28]. Phorbol 13-acetate was docked into the Unc13 structure using MOE. The structure of phorbol 13-acetate was created using ChemDraw version 13.1.1. Energy minimisation for both the protein and ligand was performed in MOE 2023, utilising the Amber 10 force field with a 0.1 RMS kcal/mol/Å^2^ gradient. The binding site for phorbol 13-acetate in *Drosophila* Unc13 was constructed around the T1731 and S1732 residues in loop 1 and G1745, I1746, and A1747 in loop 2. The binding pocket was centred on the geometry of phorbol 13-acetate as seen in the PKCδ C_1_B structure (PDB ID: 1PTR), which is homologous to our template, Munc13-1 C_1_. Molecular docking was performed using the triangle matcher placement method and rigid receptor docking, generating 50 conformer poses with 25 refinements. All docked conformers were analysed for protein-ligand interactions using Discovery Studio Visualizer 24.1.0 (DS, Biovia Inc., San Diego, CA).

#### 2.13.3 Building the phorbol 13-acetate-Unc13-membrane-ethanol complex

The protein-ligand-membrane-ethanol complex (Fig. 5C and D) was built according to the method described in our previous publication [28]. Briefly, the spatial positioning of the protein-ligand complex with respect to the lipid membrane was analysed using the PPM server. The membrane-protein-ligand complex model was built in the rectangular box using the CHARMM-GUI membrane builder web server. We used 100 % phosphatidylserine as the lipid molecule to simulate the membrane. 1 % ethanol was added to the system using GROMACS 12.4.0. The number of phosphatidylserine molecules in the upper leaflet was 64, and in the lower leaflet, 58, for both the wild type and the mutant.

## 3 Statistics

To plot data from Mendelian ratio, negative geotaxis, survival and ethanol sensitivity experiments Sigmaplot V14 (Systat) and Prism V9 or V10 (GraphPad Software) were used. The same programs were used to calculate mean, SD (standard deviation) and SEM (standard error of the mean) in negative geotaxis experiments and median, 25th, and 75th percentiles in ethanol sensitivity experiments.

Differences between Kaplan-Meier survival curves were tested for statistical significance using the Survival LogRank test in Sigmaplot V14 (SyStat). Data distribution from negative geotaxis experiments was tested for normality with a Shapiro-Wilk test. Since the data were non-normally distributed, multiple comparison analyses (groups compared to every other group) were conducted in Prism V9 or V10 (GraphPad Software) using the Kruskal-Wallis test (=One-way ANOVA on ranks) followed by Dunn's test for multiple comparisons. Data from alcohol sensitivity experiments also did not distribute normally. Therefore, the same analysis was applied, with the difference being that experimental groups were compared to one control group.

## 4 Results

### 4.1 Medical history and clinical features of the patients

#### 4.1.1 Patient 1 carries homozygous variant c.283C > T, p.(Arg95Ter)

Patient 1 was born in Syria via a caesarean section after an uneventful pregnancy. The parents are consanguineous (first and second degree cousins). Congenital arthrogryposis (distal type) was diagnosed and made conservative as well as surgical treatments necessary. Suspected atypical Ehlers Danlos syndrome (EDS) was later confirmed with the homozygous pathogenic variant NM_130468.4:c.145delG, p.(Val49Ter) in the *CHST14* gene (coding for the carbohydrate sulfotransferase 14). The boy presented at age 2.5 years with global developmental delay (supported walking and single words with 2.5 years) and microcephaly (head circumference 46.7cm/-2.2 standard deviation score) for further evaluation, as the EDS did not explain the delayed speech development. Brain sonography was inconspicuous at the age of 2 years. He uses hearing aids for mixed hearing loss. The older brother was also reported with developmental delay. We identified the homozygous nonsense variant *c.283C > T*, p.(Arg95Ter) in *UNC13C* (Fig. 1A) in the trio exome analysis of patient 1 (all variants listed in Supplemental Excel File 1). A sample from the brother was not available for genetic testing. In the last evaluation at nine years, the boy had deficits in language with a tested intelligence quotient of 87 (normal intelligence) and a borderline normal head circumference (at the age of ten years 51.0cm/-1.7 standard deviation score). Further manifestations of the *CHST14*-associated EDS comprised motor limitations, facial abnormalities, ocular and hearing problems.

#### 4.1.2 Patient 2 carries variants c.206G > T, p.(Cys69Phe) and c.956C > A, p.(Ala319Glu)

The patient was the first daughter of non-consanguineous Caucasian parents born at term of unremarkable pregnancy. In the first years of life, she presented developmental delay, especially in social skills and regression of expressive language and stereotypic movements. At 3 years of age she was diagnosed with an autism spectrum disorder according to ADOS-2 and Griffiths Mental Developmental scale. Brain MRI showed corpus callosum hyperplasia and narrowed occipital horns of lateral ventricles. Array-CGH did not reveal pathogenic copy number variants. ABR, eye examination and sleep EEG were unremarkable. At last evaluation at 10 years of age, clinical evaluation revealed normal growth and mild dysmorphisms including retrognathia and prominent incisors, tip-toe walking, stereotypies, poor and echolalic language and hetero-aggressive behaviours. The compound heterozygous variants c.206G > T, p.(Cys69Phe) and c.956C > A, p.(Ala319Glu) in *UNC13C* (Fig. 1A) were identified by whole exome sequencing on the trio.

#### 4.1.3 Patient 3 carries variants c.1642C > T, p.(Arg548Cys) and c.3311C > T, p.(Thr1104Met)

The patient is a 12-year-old female, third child of healthy non-consanguineous parents, born after an uneventful pregnancy. At the age of 14 months she was evaluated because of axial hypotonia, poor balance, global developmental delay and vertical nystagmus. At that time her occipitofrontal circumference was 43 cm (−2.5 SD). She achieved independent ambulation at 27 months of age. At nine years old, she was diagnosed with progressive sensorineural hearing loss. Physical examination revealed clumsy and unsteady movements. Her height, weight, and occipitofrontal circumference (50 cm; −1.6 SD) were within normal ranges. She experiences learning difficulties and attends a school for special education. Ophthalmologic assessment identified hypermetropia (+5.5 diopters in both eyes). She has three siblings, one of whom presented with congenital vertical nystagmus without any additional abnormalities. Brain MRI at the age of 8 months showed a cavum vergae and somewhat increased signal intensity in T2w images within the periventricular white matter. The compound heterozygous variants c.1642C > T, p. (Arg548Cys) and c.3311C > T, p.(Thr1104Met) in *UNC13C* (Fig. 1A) were identified in trio exome sequencing.

#### 4.1.4 Patient 4 carries variants c.3158C > T, p.(Thr1053Ile) and c.3566T > C, p.(Ile1189Thr)

The patient was born by primary cesarean section after an uneventful pregnancy to non-consanguineous healthy parents from Portugal. Her family history is unremarkable. She achieved independent sitting at the age of 9 months. She was referred to the genetics department at the age of 1 year and 10 months due to global developmental delay. At this age, she was able to walk but falls occurred frequently, and she babbled but did not speak clear words. Parents reported hyperactivity, a reduced attention span, impulsiveness, temper tantrums and constipation. Clinical assessment showed global developmental delay, relative microcephaly, muscular hypotonia and convergent strabismus. Morphological features included sparse temporal hair, low-set ears, mild synophrys, epicanthus, long palpebral fissures, full lips, pointed chin, sacral dimple, prominent foetal pads on fingers, bilateral brachydactyly of the fifth finger. Later she developed spastic paraparesis. Brain imaging at the age of 6 years showed hypomyelination with trigonal emphasis, as well as hypoplasia of the pons and the superior vermis. Chromosomal microarray analysis was inconspicuous. The compound heterozygous variants c.3158C > T, p.(Thr1053Ile) and c.3566T > C, p.(Ile1189Thr) in *UNC13C* (Fig. 1A) were identified in trio exome sequencing.

### 4.2 Additional patients with *UNC13C* variants, not recreated in *Drosophila*

Here, we present seven more cases with variants in the *UNC13C* gene and neurodevelopmental impairments. These variants, however, were not recreated and analysed in *Drosophila*, since they were identified at an already advanced stage of the experimental part of the project. They may be subjects of a future study.

#### 4.2.1 Patient 5 carries the homozygous variant c.4301G > A, p.(Cys1434Tyr)

Trio exome sequencing of a foetus with multiple severe brain malformations identified a homozygous missense variant of unknown significance in *UNC13C* c.4301G > A, p.(Cys1434Tyr). The malformations were observed sonographically at 22 weeks of gestation and included agenesis of the corpus callosum, cerebellar vermis hypoplasia, dilated lateral ventricles up to 18–20 mm, and hydrocephalus. The Israeli Arab parents were consanguineous and reported a previously terminated pregnancy involving a foetus with similar brain malformations. In addition, they have three reportedly healthy children. This *UNC13C* variant affects an evolutionarily conserved residue and is *in silico* predicted as deleterious (Supplemental Excel File 1). Notably, there were no alternative candidate variants to explain the phenotype.

#### 4.2.2 Patient 6 carries the homozygous variant c.1150C > A, p.(Pro384Thr)

Patient 6 is a boy of consanguineous parents of Arabic descent who presented with global developmental delay, ataxia, oculomotor apraxia, dysgenesis and polymicrogyria of the cerebellar hemispheres and vermis. His speech development seemed initially delayed, but he caught up well, allowing regular schooling. Trio exome sequencing revealed the homozygous variant c.1150C > A, p.(Pro384Thr) in *UNC13C*. His brother, who also presented with delayed motor development but no oculomotor apraxia, carries a different *de novo* pathogenic variant explaining his phenotype, which was not identified in patient 6. Further segregation of the variant in the family was not possible. This *UNC13C* variant affects an evolutionarily moderately conserved residue and is *in silico* predicted as likely benign (Supplemental Excel File 1).

#### 4.2.3 Patient 7 carries variants c.692G > A, p.(Ser231Asn) and c.4234C > T, p.(Arg1412Cys)

Patient 7 was referred for genetic testing due to severe language delay and joint hypermobility. She was born to non-consanguineous, healthy parents from Laos. Her family history showed a language delay in her father and her paternal half-brother with consecutively normal psychomotor development. At the age of eight, she went to a special education school. Chromosomal microarray analysis showed a paternally inherited deletion of unknown significance of 288 kb (chr4:163924142-164211730) for which segregation analysis was not performed. The compound heterozygous variants c.692G > A, p. (Ser231Asn) and c.4234C > T, p.(Arg1412Cys) in *UNC13C* were identified by trio exome sequencing. *In silico* prediction is not informative for the first variant and predicts a deleterious effect for the second variant.

#### 4.2.4 Patient 8 carries variants c.283C > T, p.(Arg95Ter) and c.3789G > C, p.(Leu1263Phe)

The patient is a 9-year-old son of non-consanguineous parents of Turkish, French and Austrian origins. He was born at full term of an uneventful pregnancy and was diagnosed at birth with an imperforate anus. His renal ultrasound and echocardiogram were normal. He was referred to the medical genetics department for language delay which persisted despite the treatment of glue ears. He first spoke in sentences at the age of 5 years and still has difficulties with understanding complex instructions and constructing correct sentences. He attends a regular school with a personal assistant. A cerebral MRI performed at the age of 8 years was normal. Hypertrichosis was noted from the age of 6 years, without indications for an early puberty. He wears glasses for a mild myopia. The variants in *UNC13C* were identified by genome sequencing and are in a compound heterozygous state. The nonsense variant c.283C > T, p.(Arg95Ter) is the same as in patients 1 and 9–11.

#### 4.2.5 Patients 9–11 carry homozygous variant c.283C > T, p.(Arg95Ter)

Patients 9–11 were from the same family, all presenting with a neurodevelopmental phenotype. Patient 9 was a 6-year-old boy at the time of testing. He presented with intellectual disability, speech delay, motor delay, hypotonia, asthenia, dysmetria, and gait ataxia. He also had severe eczema, camptodactyly, and delayed myelination. Exome sequencing revealed no diagnostic finding, identified a homozygous nonsense variant, c.283C > T, p.(Arg95Ter), in *UNC13C* (the same mutation as patient 1 and 8). This homozygous variant was inherited from each of the parents, who were heterozygous. Patient 10 was the elder brother to Patient 9. He was 9 years old at the time of testing, with similar clinical features as his brother. His exome also revealed no diagnostic finding but identified the same familial homozygous nonsense variant in *UNC13C*. Patient 11 was from the same family, the younger sister to Patients 9 and 10 and carries the same mutation. She presented with similar features as her siblings and was 1 year old at the time of testing. Additionally, exome sequencing revealed a *de novo* likely pathogenic heterozygous variant, c.304C > T, p.(Gln102Ter), in *CSNK2B*. Pathogenic variants in *CSNK2B* are associated with autosomal dominant Poirier-Bienvenu neurodevelopmental syndrome [29]. This variant was predicted to cause loss-of-function of the gene, which is consistent with the disease mechanism [30].

### 4.3 Possible pathogenicity of variants and generated knock-in fly strains

The homozygous variant in the codon encoding an arginine (R95) of patient 1, 8, 9, 10 and 11 (Fig. 1A) leads to the creation of a stop codon. This nonsense mutation results in a heavily truncated product containing less than 100 amino acids devoid of all known functional domains; additionally, given the early stop codon, these transcripts may be subject to nonsense-mediated decay. We assumed that this homozygous allele combination is most probably equivalent to a homozygous null mutation, which is lethal when introduced into *Drosophila unc13* [1]. Therefore, it was not analysed further.

Patients 2, 3, and 4 carried two different variants each (Fig. 1A). There are no accounts of these variants in a homozygous state in the human reference population database (gnomAD v4) [31], except variants #1 and #4, each with one homozygous allele entry. Variants #2 and #3 of patient 2 are located in the N-terminal region (Fig. 1A). Variant #2, *unc*^*#2C69F*^ replaced a cysteine with phenylalanine in patient 2. Although cysteine substitutions might be considered likely to cause functional defects due to their role in forming disulfide bridges, a pathogenic mechanism in this case remains elusive, as we are not aware of this specific residue participating in such bonds. Additionally, this position is not conserved between *Drosophila* and human (Fig. 1B). A possible explanation for the potential pathogenicity of the variant #3 remains elusive. Variant #4, *unc*^*#4R548C*^ of patient 3 is also part of the unstructured N-terminal region and is located adjacent to a phosphorylation site, which may be impacted by the variant. In contrast, variant #5, i.e., *unc13*^*T1104M*^*,* is localised in the C-terminal C_1_ domain (Fig. 1A). Variant #5, in particular, was hypothesised to induce damage to the protein since the position is conserved between human and *Drosophila* (Fig. 1B). Additionally, previous studies in mice proved the importance of the C_1_ domain of Munc13-1 for synaptic efficacy and survival [32]. Variants #6 and #7 of patient 4 flank the C_1_ domain (Fig. 1A).

Out of the six analysed variants, only positions of variants #5 and #7 are conserved between fly and human. This fact adds complication when interpreting results. In all of the variants in humans an amino acid was exchanged for an amino acid with different chemical properties, while some corresponding mutations in *Drosophila* are inert. We sum up the human variants and the mutations introduced in *Drosophila* and the properties of the amino acids in Table S9. Nevertheless, we generated transgenic *Drosophila* strains for all of the six variants, including those not conserved, for two reasons. First, the corresponding positions in *Drosophila* may still play a critical role in gene function despite the lack of conservation. Second, to further evaluate the efficiency of the *ovo*^*D*^/CRISPR-Cas9 mutagenesis protocol previously used [6], now on a larger scale. In this previous technical study concerning the optimal implementation of the scarless *ovo*^*D1*^-assisted CRISPR/Cas9 pipeline, we successfully created alleles that reconstruct patient variants #2 and #3 in *Drosophila*. Here, we applied the same approach to recreate variants #4, #5, #6 and #7. In total, 22 individual strains containing one of the six mutations were generated alongside two negative control strains without any amino acid mutation (Table S4), showcasing the mutagenesis protocol's efficiency. It would have been feasible to create ten *Drosophila* strains per mutation from one injection round if necessary.

### 4.4 Crosses and control experiments

*Drosophila* harbours one *unc13* gene only. Homozygous *unc13* null mutations are lethal as embryos show no muscular peristalsis or coordinated movement required for hatching and die in late embryonic stages [1]. Crossing an *unc13*^*X*^ point mutation and a null mutant allele into one animal and determining the Mendelian ratios should, therefore, directly assess the lethality of the point mutation since it is not compensated by a wild-type allele. *unc13*^*X*^*/ci*^*D*^ flies, which carry the point mutation '*X*', i.e. #2, 3, 4, 5, 6, or 7 on chromosome 4 and the dominant phenotypic marker *ci*^*D*^ on the other 4th chromosome (*in trans*) were crossed with *unc13*^*KO*^/*pan*^*2*^ flies, i.e., an *unc13* null allele over the dominant phenotypic marker *pan2*. The *unc13* null mutation is caused by the insertion of an *ActinGFP* transposon, including a *w*^*+ mC*^ element, into the *unc13* locus. Larvae with this insertion show green fluorescing signals in mid-gut muscles. Additionally, adults have red eyes instead of white eyes. This way, all four possible genotypes can be phenotypically discriminated in adult flies (Fig. 2A). The Mendelian ratios of the F1 generation adult flies were quantified. The *ci*^*D*^ allele encodes a dysfunctional Ci:Pan fusion protein, and this defect is incompatible with the loss of function caused by the *pan*^*2*^ mutation. Consequently, no, or very few, adult flies of the *ci*^*D*^*/pan*^*2*^ genotype were expected to eclose. Thus, 33.3 % of the remaining genotypes were expected if no impact on viability was caused by an engineered *unc13*^*X*^ allele (Fig. 2A). To assess a possible haploinsufficient or dominant negative effect of *unc13*^*KO*^ or *pan*^*2*^, which would complicate the analysis of Mendelian ratios, we crossed *unc13*^*KO*^*/pan*^*2*^ to *w*^*1118*^. A 50/50 distribution between *unc13*^*KO*^*/+* and *pan*^*2*^*/+* was expected if neither *unc13*^*KO*^ nor *pan*^*2*^ had a dominant negative effect (Fig. S1). The obtained ratio was very close to the expected, and we concluded that neither *unc13*^*KO*^ nor *pan*^*2*^ is haploinsufficient or exerts a dominant negative effect. A possible haploinsufficient or dominant negative effect of *unc13*^*X*^ or *ci*^*D*^ could be excluded indirectly from other experiments.

Additionally, to test for possible adverse effects of the mutagenesis procedure itself [6], we subjected embryos to the same transgenesis procedure but with plasmids carrying silent mutations. To create the first negative control genotype (*Ctrl 1*), mutagenesis involved cutting the *unc13* locus at CRISPR sites I and II (Fig. 1A) followed by homology-directed repair (HDR) with a vector, which carried silent mutations, to readily identify successful recombinants via sequencing. The resulting fly stocks served as negative control for mutations #2, #3, #4 (Fig. 1A). Similarly, a second control genotype (*Ctrl 2*) was generated for the mutations #5, #6, and #7 (Fig. 1A). The measured Mendelian ratios for both negative control genotypes were very close to the expected ones (Fig. S2). Thus, the mutagenesis procedure did not introduce significant undesired on- or off-target effects. These results also show that one *ci*^*D*^ has no dominant negative effect; otherwise, the fraction of *unc13*^*KO*^*/ci*^*D*^ should be reduced.

### 4.5 Mendelian ratio experiments show no clear negative effect of mutations

#### 4.5.1 Patient 2

Five individual fly strains were generated for mutation #2 and five for mutation #3 (Table S4), both found in patient 2*.* Here, the homologous positions in *unc13* of *Drosophila* were mutated to phenylalanine (F) and glutamate (E), respectively (Fig. 1B). Fly crosses and resulting Mendelian ratios in the first filial generation (F1) did not show an effect of either of the two mutations when crossed over *unc13*^*KO*^ (Fig. 2A and B). To test whether combining mutations #2 (strain 1) and #3 (strain 1) to simulate the genotype of patient 2, we crossed *unc13*^*#2*^*/ci*^*D*^ with *unc13*^*#3*^*/ci*^*D*^. The expected Mendelian ratios are shown in Fig. 3A. No negative effect of the *trans*-heterozygosity of this allele combination was detected (Fig. 3B).

#### 4.5.2 Patient 3

Patient 3 carries the variants #4 and #5 (Fig. 1A). Position of the variant #5 is conserved between human and *Drosophila* and is located in the C_1_ domain. In an experiment analogous to the one described before, *unc13*^*#4*^*/ci*^*D*^*, or unc13*^*#5*^*/ci*^*D*^ were crossed with *unc13*^*KO*^*/pan*^*2*^. Since the previous experiments have shown very consistent results between the different fly strains of the same genotype, we decided to keep only three strains of mutation #4 and three strains of mutation #5. Both mutations showed consistently no negative effect on the observed Mendelian ratios when crossed over *unc13*^*KO*^ (Fig. 2C). The combination of mutation #4 (strain 1) and #5 (strain 1) to simulate the genotype of the patient also presented itself as harmless (Fig. 3B).

#### 4.5.3 Patient 4

The patient carries variants #6 and #7 (Fig. 1A). Mendelian experiments were conducted in *Drosophila* as described for the patients 2 and 3 above. None of the three strains of mutation #6 showed a negative effect when crossed over *unc13*^*KO*^ (Fig. 2D). Strains 1 and 2 of mutation #7 also showed a similar result (Fig. 2D). However, when strain 3 of mutation #7 was crossed with *unc13*^*KO*^*/pan*^*2,*^ no *unc13*^#7^/*unc13*^*KO*^ hatched (Fig. 2D), indicating a cardinal damage to the *unc13* gene. In a previous publication, we showed that our improved CRISPR/Cas9 mediated mutagenesis approach induces undesired deletions or insertions in rare cases, leading to a shift in the reading frame [6]. Since strains 1 and 2 of mutation #7 showed no effect, we assumed an undesired mutation in strain 3, and, therefore, excluded this strain from the analysis. Simulating the genotype of the patient by crossing *unc13*^#6^ (strain 3) over *unc13*^#7^ (strain 2) showed no negative effect on the Mendelian ratios (Fig. 3B). The strains utilised in this experiment were selected exclusively based on the availability of a high number of virgin females and males.

### 4.6 Negative geotaxis experiments reveal no apparent effect of the mutations

Next, we assessed a behavioural trait of the mutants – negative geotaxis – as it might be a more sensitive readout than the Mendelian experiments (Fig. 4A). This behavioural paradigm is based on the natural impulse of flies to climb or fly upwards against gravity. Behaviours like these involve entire neuronal circuits and several neuronal functions, including proprioception, central processing, and motor neuron activation. Therefore, the compound effect of *unc13* mutations on many synapses can be measured. Since there is only one *unc13* gene in the fly genome and since Unc13 plays a role at central and peripheral synapses [1,14,33,34], this behavioural assay might reveal a deficit of the mutated Unc13 protein.

To test this hypothesis, we crossed *unc13* point mutations with *unc13*^*KO*^ to create *unc13*^*X*^/*unc13*^*KO*^ and *unc13*^*X*^ over *unc13*^*Y*^ to recreate the genotypes *unc13*^*X*^*/unc13*^*Y*^ of patients 2, 3, and 4. As a positive control, we used a null mutant of the *Drosophila* gene for the adhesion GPCR *Latrophilin/Cirl* (*Cirl*^*KO*^, labelled ‘pos. Ctrl’) [35] for which we previously found a robust and reproducible phenotype in this assay (unpublished data). We routinely use this line as a positive control. It shows a robust, but not too strong phenotype, allowing to assess the reliability of the assay. This positive control showed a sizable difference when compared to both negative control genotypes, *unc13*^*Ctrl 1*^*/unc13*^*KO*^ and *unc13*^*Ctrl 2*^*/unc13*^*KO*^. However, none of the mutations differed significantly from the respective negative control genotypes (Fig. 4B–Tables S6 and S7).

### 4.7 Lifespan assays do not reveal a clear effect of the mutations

Next, we tested the impact of *unc13* mutations on lifespan. Therefore, 16 flies were isolated in vials to observe their lifespans and to derive Kaplan-Meier survival curves. When we generated *unc13*^*X*^/*unc*^*KO*^ or the patient genotypes *unc13*^*X*^/*unc13*^*Y*^, we consistently observed significant overlap between the survival curves of the genotype of interest and the respective negative control genotypes (*unc13*^*Ctrl 1*^/*unc*^*KO*^ or *unc13*^*Ctrl 2*^/*unc*^*KO*^). Survival LogRank tests revealed no statistically significant differences between these survival curves (Fig. 4C,D,E). The only genotype with a tendency towards a statistical difference was the patient 3 genotype unc13^#4^/unc13^#5^, with p-values of 0.089 and 0.059 when compared to the two control genotypes.

### 4.8 Three mutations show a pronounced distortion in the alcohol sensitivity behaviour

Ethanol has long been understood to inhibit presynaptic activity [36]. Previously, we showed that crossing the *unc13* null mutants (*unc13*^*KO*^) with a wild-type allele of *unc13* to generate heterozygous *unc13*^*KO*^*/+* flies resulted in increased resistance to the behavioural and synaptic effects of alcohol compared to wild-type flies [37]. These results indicate that *unc13* exhibits haploinsufficiency with respect to normal ethanol sensitivity, and reducing these proteins mimics a more resistant state. To investigate the effects of mutations #2 - #7 on alcohol behavioural sensitivity, we examined each mutation as a homozygote and compared them to the relevant background controls using the Loss-of-Righting Reflex (LoRR) alcohol sedation assay. Here, flies were exposed to alcohol vapour (Fig. 5A), and the time when 50 % of flies lost their righting reflex, defined as the inability to stand up after gentle tapping of the vial, was registered. Notably, *unc13*^*#2*^ and *unc13*^*#3*^ displayed significantly increased t_1/2_ LoRR scores, indicating greater alcohol resistance than the respective control (Fig. 5B–Table S8). Both mutations are located in an unstructured protein region (Fig. 1A) and are limited to the *A* isoform of *unc13*. Conversely, *unc13*^*#5*^ flies exhibited increased alcohol sensitivity (Fig. 5B–Table S8). This mutation is localised in the C_1_ diacylglycerol-binding domain of both *A* and *B* Unc13 isoforms. Ethanol is known to bind to the C_1_ domain at physiological concentrations, inhibiting diacylglycerol binding [37].

### 4.9 Molecular docking simulation reveals structural changes in the C_1_ domain of Unc13^T1729M^

To understand the reason behind the changed alcohol sensitivity in the mutants on a molecular structural level, we turned to molecular dynamics simulations. Since mutations *unc13*^*#2*^ and *unc13*^*#3*^ are located in unstructured regions, only *unc13*^*#5*^ was analysed as it is located at the activator binding C_1_ domain. To this end, a homology model for the C_1_ domain of *Drosophila* Unc13 was generated (Fig. 5C, inset) using the rat Munc13-1 C_1_ NMR structure. The modelled structure of *Drosophila* Unc13C_1_ and the template structure, Munc13-1 C_1_, showed the root mean square deviation (RMSD) with an acceptable value of 0.982 Å. In the docked structure, phorbol 13-acetate (Fig. 5C and D, green) formed a hydrogen bond with W1744 in wild-type Unc13C_1_, but it formed a hydrogen bond with R1784 in the T1729 M mutant. The binding energy of phorbol ester with the wild-type *Drosophila* Unc13C_1_ and the mutant was −4.21 kcal/mol and −4.36 kcal/mol, respectively.

Structural analysis of the protein-ligand-membrane-ethanol complex revealed that the tilt angle, defined by the orientation of the protein in the membrane, and the depth/hydrophobic thickness, defined as the depth of protein penetration in the lipid membrane, were significantly different for the wild-type and the mutant protein (Table 1). In the presence of ethanol (Fig. 5C and D), the tilt angle changes were much higher for the mutant (35°) than the wild type (5°). Further, in the presence of ethanol, the depth of penetration of wild-type Unc13C1 was 3.4 ± 2.4 Å, while in the mutant, it was 2.1 ± 0.8 Å.

**Table 1:** Structural parameters of wild-type *Drosophila* Unc13C_1_ and Unc13C_1_^T1729M^ in phospholipid membrane.

	Wild-type Unc13C1		MutantUnc13C1T1729 M	
EtOH	-	+	-	+
Tilt angle (^o^)	131 ± 11	126 ± 14	47 ± 11	82 ± 5
Depth/hydrophobic thickness (Å)	5.4 ± 2.0	3.4 ± 2.4	3.7 ± 2.3	2.1 ± 0.8
ΔG transfer (kcal/mole)	−4.1	−2.9	−3.9	−3.9
Membrane-embedded residues	P1733, Y1735, L1742, & I1746	P1733, Y1735 & L1742	P1733, & Y1735, L1742	L1726, L1727 & M1729

The ΔG-transfer, or Gibbs free energy, indicates the system's stability, with lower values signifying greater stability. For wild-type Unc13C_1_, the free energy was −4.1 kcal/mol; however, adding ethanol destabilised the system. In contrast, adding ethanol to the mutant did not change the stability of the system. The membrane-embedded residues in the presence and the absence of ethanol are shown.

## 5 Discussion

The main objective of this study has been to investigate whether the compound heterozygous variant pairs identified in patients 2, 3 and 4 are causally linked to the observed neurodevelopmental delays. Our analyses, including Mendelian ratio, negative geotaxis and lifespan experiments, provided no evidence to support this hypothesis. In patient 2, the positions of both variants are not conserved between *Drosophila* and humans (Fig. 1B), which might explain the absence of phenotypic effects in the fly model. Another straightforward explanation is that both mutations of patient 2 introduced in *Drosophila* are inert. Similarly, the position of variant #4 of patient 3 is also not conserved, which again might explain the lack of an effect in *unc13*^*#4*^*/unc13*^*KO*^ and *unc13*^*#4*^*/unc13*^*#5*^. However, the position of variant #5 of the same patient is conserved (Fig. 1B). Considering that mutation #5 is located within a functional domain, an effect in the experimental paradigms was anticipated in *unc13*^*#5*^*/unc13*^*KO*^, however, it was not observed. We found comparable results for variants #6 and #7 of patient 4: the position of #6 is not conserved, while the position of #7 is conserved, again with no detectable defect in any allelic configuration. It may be the subject of future studies whether some of the further variants presented here (patients 5, 6, 7, 8), but not analysed in *Drosophila* yet, might substantiate the potential role of *UNC13C* in neurodevelopmental disorders in humans.

One of the objectives of the present study has been to evaluate the efficiency of the previously used *ovo*^*D*^/CRISPR method [6] for generating scarless point mutations in *Drosophila,* now on a larger scale. We successfully established 22 strains of six different mutations of the *unc1*3 gene. Mendelian experiments (Fig. 2B and C,D) suggest that, while the results are generally consistent across fly strains of the same genotype, second site hits, with a significant impact on protein function, can still occur, as observed in strain 3 of mutation #7 (Fig. 2 D). Consequently, generating three to five fly strains per SNV is recommended. A typical injection round of 200 embryos was generally sufficient to produce over ten strains of the same mutation. Acknowledging the genetic and anatomical differences between *Drosophila* and humans, the presented strategy may prove useful for large-scale analyses of the pathogenicity of variants with uncertain significance.

It has long been established that physiologically relevant ethanol concentrations inhibit the activity of presynaptic terminals [36] and that Unc13 proteins are likely mediators of this effect [37,38]. In this study, we tested the six variants of patients 2, 3 and 4 in a homozygous configuration, as all mutants were viable as homozygotes. Previously, we reported that *unc13*^*KO*^ heterozygotes displayed resistance to alcohol sedation [37]. Notably, *unc13*^*#2*^ and *unc13*^*#3*^ homozygotes exhibited a remarkable increase in alcohol resistance, with the t_1/2_ LoRR (median) extended by 5.0 and 6.6 min, respectively (Fig. 5B), closely mirroring the 7 min increase previously observed in *unc13*^*KO*^*/+* [37]. Hence, these point mutations are likely to affect Unc13 function and, as homozygotes, produce effects similar to a haploinsufficiency for both Unc13A and B isoforms. In *unc13*^*#2*^, a valine was substituted by phenylalanine in an unstructured region (Fig. 1), both of which are hydrophobic amino acids. In *unc13*^*#3*^, an aspartate residue was replaced by glutamate, again in an unstructured region (Fig. 1). These amino acids also share similar properties, leaving the underlying mechanism unclear. Conversely, *unc13*^*#5*^ homozygotes, carrying a point mutation in the C_1_ domain, had the opposite effect, increasing alcohol sensitivity with a 3-min reduction in LoRR compared to controls (Fig. 5B). This result also strongly suggests that this mutation has a measurable effect on Unc13 activity, which might be expected, given our previous findings that ethanol binds to the C_1_ domain, inhibiting synaptic vesicle release. Here, molecular dynamics simulations provided further insight into the possible mechanism underlying this effect.

The binding of DAG/phorbol ester at the C_1_ domain of Unc13 and its association with the plasma membrane is the primary step of vesicle fusion. In the present study, we simulated a phorbol 13-acetate-Unc13-C_1_-membrane complex. Our molecular dynamics simulation results showed that mutation #5 (T1729 M) caused major changes in the tilt angle and the depth of membrane penetration of Unc13. Ethanol caused additional structural changes but ultimately stabilised the protein-ligand-membrane system in the mutant as compared to the wild type protein. The ethanol molecule with its hydroxyl group can form hydrogen bonds, and with the small lipophilic chain can exert hydrophobic interactions. If we just consider the protein-ethanol complex, there is a possibility that mutating the threonine residue which has higher propensity of forming a hydrogen bond with the ethanol molecule, to a more hydrophobic methionine residue would reduce the alcohol binding of the mutant protein. However, Dunc13 is a peripheral membrane protein, and we should consider the effect of ethanol in the context of the protein-ligand-membrane system. Our results show that in the presence of ethanol, the tilt angle changes were much higher for the mutant (35°) than the wild type (5°) and showed a lower value of the depth of penetration in the membrane (2.1 ± 0.8 Å in the mutant versus 3.4 ± 2.4 Å in the wild-type protein). These changes bring the methionine residue of the mutant protein closer to the membrane but do not completely embed it inside the membrane. The PPM server, which we used to analyse protein alignment within the membrane, considers a residue as an embedded one even with minimal contact (when even a single atom touches the membrane). In our case, residue M1729 is located precisely at the membrane interface, enabling methionine to interact simultaneously with both ethanol and the membrane. With this orientation the hydrophobic side chain of methionine interacts with the –CH_2_CH_3_ group of the ethanol molecule showing higher ethanol binding and sensitivity. For wild type Dunc13C_1_, the free Gibbs energy (ΔG) was −4.1 kcal/mol. The addition of ethanol destabilised the system (ΔG = −2.9 kcal/mol). In contrast, adding ethanol to the T1729M-ligand-lipid did not affect the stability of the system (ΔG = −3.9 kcal/mol, unchanged). There could be many factors contributing to the value of ΔG transfer, which cannot be fully explained by changes in tilt angle or hydrophobic thickness or by ethanol binding alone. However, the final value of ΔG indicates that protein-ligand-membrane-ethanol system is more stable for the mutant (−3.9 kcal/mol) than the wild type (−2.9 kcal/mol). We believe this is the case in T1729 M mutant flies (*unc13*^*#*5^) that showed higher ethanol sensitivity in experiment than the wild type flies. It is important to mention here that we used 1 mol% or 555 mM ethanol concentration for our modelling studies. This value is about 3 times the concentration to induce anaesthesia in tadpoles [39], 30 times above the legal threshold for intoxication in humans [40] and ∼2 times the concentrations in the fly hemolymph following intoxication [41]. Although the concentration was rather high, this concentration was used earlier by several groups for modelling and simulation studies [28,42,43]. Furthermore, our data is based on modelling only the C_1_ domain, and it is expected that domain-domain interactions in the full-length Dunc13 would alter the magnitude of ethanol sensitivity. Earlier, we reported an alcohol binding site in the C_1_ domain of Munc13-1 [37,38]. Our current results with *Drosophila* Unc13 further highlight the importance of the C_1_ domain in modulating ethanol sensitivity. Moreover, the findings might inspire future investigations concerning the pharmacological modulation of synaptic activity, possibly in the context of neurodevelopmental disorders. Further, it is intriguing to speculate that variation in *UNC13* genes contributes to differing ethanol sensitivity in humans. However, to our knowledge, no extensive studies have investigated this aspect.

## Author contributions

D.L., A.M., T.L., and R.A.J. conceived and supervised the study; D.L., A.M. prepared the figures and wrote the manuscript with consent from all co-authors.

Conceptualisation D.L., A.M., T.L., R.A.J., G.R., G.S.

Methodology F.M., D.L., A.M., T.L., R.A.J., G.R., G.S, J.D., N.P.N., A.K.D., N.S.

Validation D.L., A.M., F.M., S.N., G.S., N.P.N., J.D., G.R.

Formal analysis D.L., A.M., F.M., S.N., G.S., N.P.N., J.D., G.R.

Investigation F.M., S.N., G.S., N.P.N., J.D., T.S.B., N.B.-P., G.T., A.B., O.P., J.M., K.S., E.B., K.L.vG., A.Re., K.J.G., A.Ra, J.R., D.F., J.A.R., M.H.K.C., E.M., S.S., X.L., A.K.D., N.S., G.R., T.L. R.A.J., A.M., D.L.

Writing – Original draft D.L., A.M.

Writing – Review & Editing F.M., S.N., A.M., G.S., N.P.N., K.J.G., A.K.D., N.S., J.D., G.R., T.L., R.A.J., D.L., T.S.B., N.B.-P., A.B., O.P, J.M., A.Re., K.L.vG., E.B., K.S., G.T., N.B.-P., A.Ra, J.R., D.F., O.P., J.A.R., M.H.K.C.

Visualization F.M., D.L., T.L., G.S., G.R.

Supervision D.L., A.M., T.L., R.A.J., G.S., G.R.

Project administration T.L., R.A.J.

Funding Acquisition D.L., A.M., T.L., T.S.B., N.S., S.N., R.A.J., J.D., G.R.

## Declaration of generative AI and AI-assisted technologies in the writing process

The authors used the free online versions of ChatGPT V3.5, Deepseek V3 and the commercial version of Grammarly to improve the manuscript's language and readability. The authors thoroughly reviewed and edited the content and take full responsibility for the final version of the publication.

## Funding

This work was supported through the "Junior Research Grant" from the Medical Faculty of 10.13039/501100008678Leipzig University to D.L. and N.S., by the "Clinician Scientist Program" of 10.13039/501100008678Leipzig University and the 10.13039/501100004038Jung Foundation for Science and Research through "Jung Career Advancement Prize" 2023 to A.M.; K.J.G. was funded by the "Promotionsförderung" program of the Medical Faculty of Leipzig University; T.S.B. was supported by the 10.13039/501100003246Netherlands Organisation for Scientific Research (10.13039/501100001826ZonMw Vidi, grant 09150172110002); This work was in part supported by the 10.13039/100031478NextGenerationEU grant ‘Rafforzamento e potenziamento della ricerca biomedica del SSN’ (PNRR-MR1-2022-12376412 to N.B.P.) and was generated within the European Reference Network for Rare Malformation Syndromes, Intellectual and Other Neurodevelopmental Disorders (ERN ITHACA); This work was further supported by grants from the 10.13039/501100001659Deutsche Forschungsgemeinschaft (DFG) to N.S. through CRC1423 project B06 (project number 421152132); S.N. and R.A.J. are supported by the 10.13039/501100001659Deutsche Forschungsgemeinschaft (537144118537144118 with AB393/9-1 and NE2706/2-1); This work was also supported by the 10.13039/100000002National Institutes of Health Grant AA022414-01 to J.D. and G.R.

The open access publishing fee was covered under the agreement between DEAL Consortium and Elsevier.

## Declaration of competing interest

The Department of Molecular and Human Genetics at Baylor College of Medicine receives revenue from clinical genetic testing completed at Baylor Genetics Laboratories.

## Data Availability

Raw data of identified *UNC13C* variants with overview of information from different public databases and of all *UNC13C* variants from the database gnomAD v4.1.0 (https://gnomad.broadinstitute.org/) are available in the Supplementary Excel file. Other raw data are available from the corresponding authors on request. Computer Code has not been generated.
