# Supplementary material for: Single nucleotide variants in UNC13C associated with neurodevelopmental disorders affect ethanol sensitivity in Drosophila
Source: Biochem Biophys Rep. 2025 Nov 28;45:102375. doi: 10.1016/j.bbrep.2025.102375 (PMC12702192; doi:10.1016/j.bbrep.2025.102375)
Supplement: Multimedia component 2 [file mmc2.docx]

# SUPPLEMENTAL MATERIAL

**Müller et al.**, “Single nucleotide variants in *UNC13C* associated with neurodevelopmental disorders affect ethanol sensitivity in *Drosophila”*


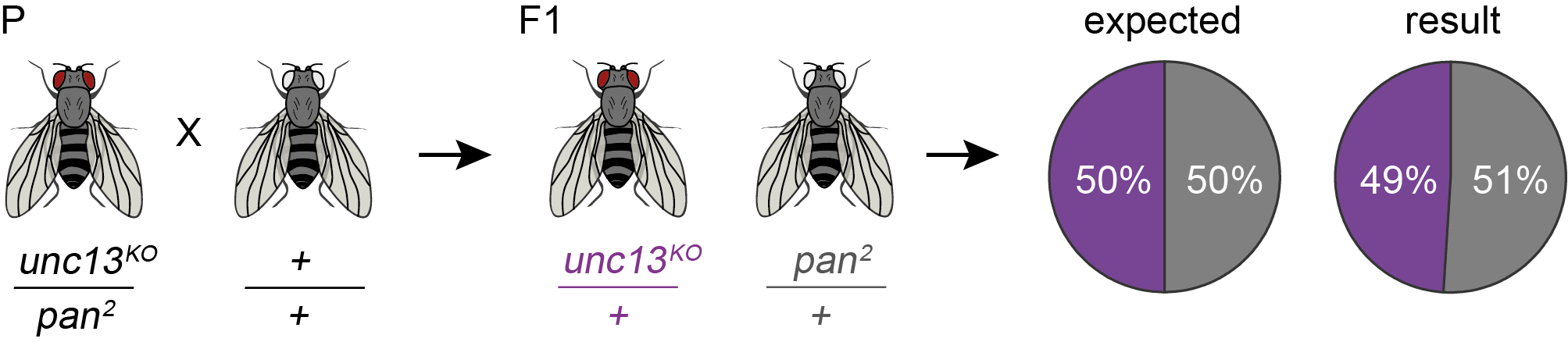


**Figure S1. Mendelian experiments indicate no haploinsufficient or dominant negative effect of unc13^KO^ or pan^2^.**

Crossing scheme and expected Mendelian ratio in the experiment designed to assess whether unc13^KO^ or pan^2^ exhibit haploinsufficient or dominant negative effects. unc13^KO^/pan^2^ flies were crossed with w1118 flies (+/+). P, parental generation; F1, first filial generation. If neither unc13^KO^ nor pan^2^ exerts haploinsufficient or dominant negative effects, the expected phenotypic (eye colour) ratio in F1 is 50/50. unc13^KO^/+, violet; pan^2^/+, grey.


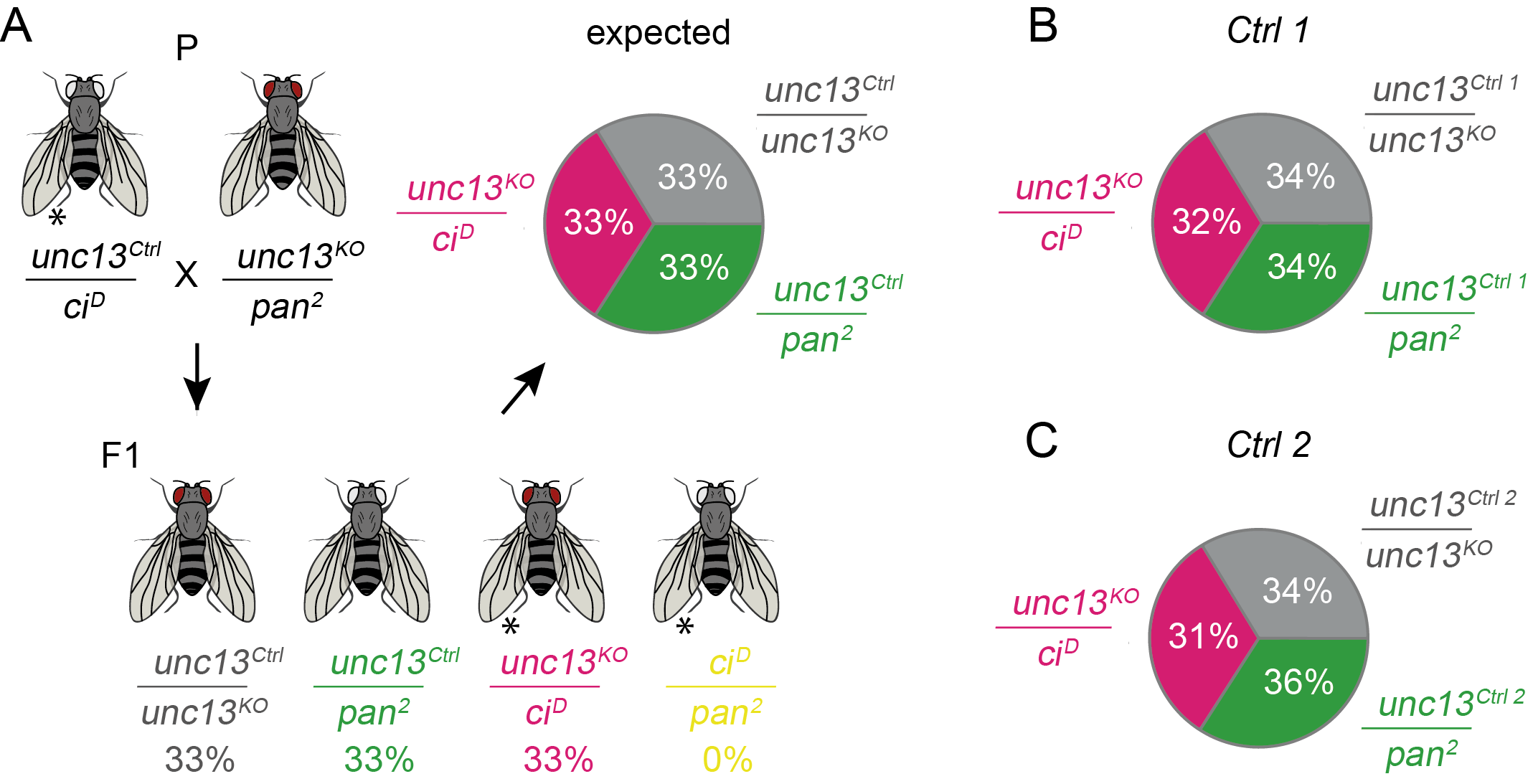


**Figure S2. Mendelian experiments shows no negative effect of the CRISPR-based mutagenesis procedure.**

**(A)** Crossing scheme and expected Mendelian ratio in experiments designed to test whether the CRISPR procedure introduces systematic unwanted negative effects on the gene product. Silent mutations were introduced to assess this. P, parental generation; F1, first filial generation. F1 genotypes can be identified using dominant phenotypic markers: Red eyes indicate the presence of unc13^KO^, and a shortened wing vein (marked with *) indicates the presence of ci^D^. unc13^Ctrl 1 or 2^/unc13^KO^, grey; unc13^Ctrl 1 or 2^/pan^2^, green; unc13^KO^/ci^D^, magenta. The allele combination ci^D^/pan^2^ (yellow) is lethal and should not be observed among hatched flies. If the control mutations unc13^Ctrl 1^ and unc13^Ctrl 2^ have no impact, the expected distribution is 33.3% for each viable genotype.

**(B, C)** Mendelian ratios for unc13^Ctrl 1^ and unc13^Ctrl 2^. (B) A total of 270 flies were counted. (C) A total of 213 flies were counted. Related to Figure 2 of the main text.

Due to rounding, percentages do not always add up to 100%


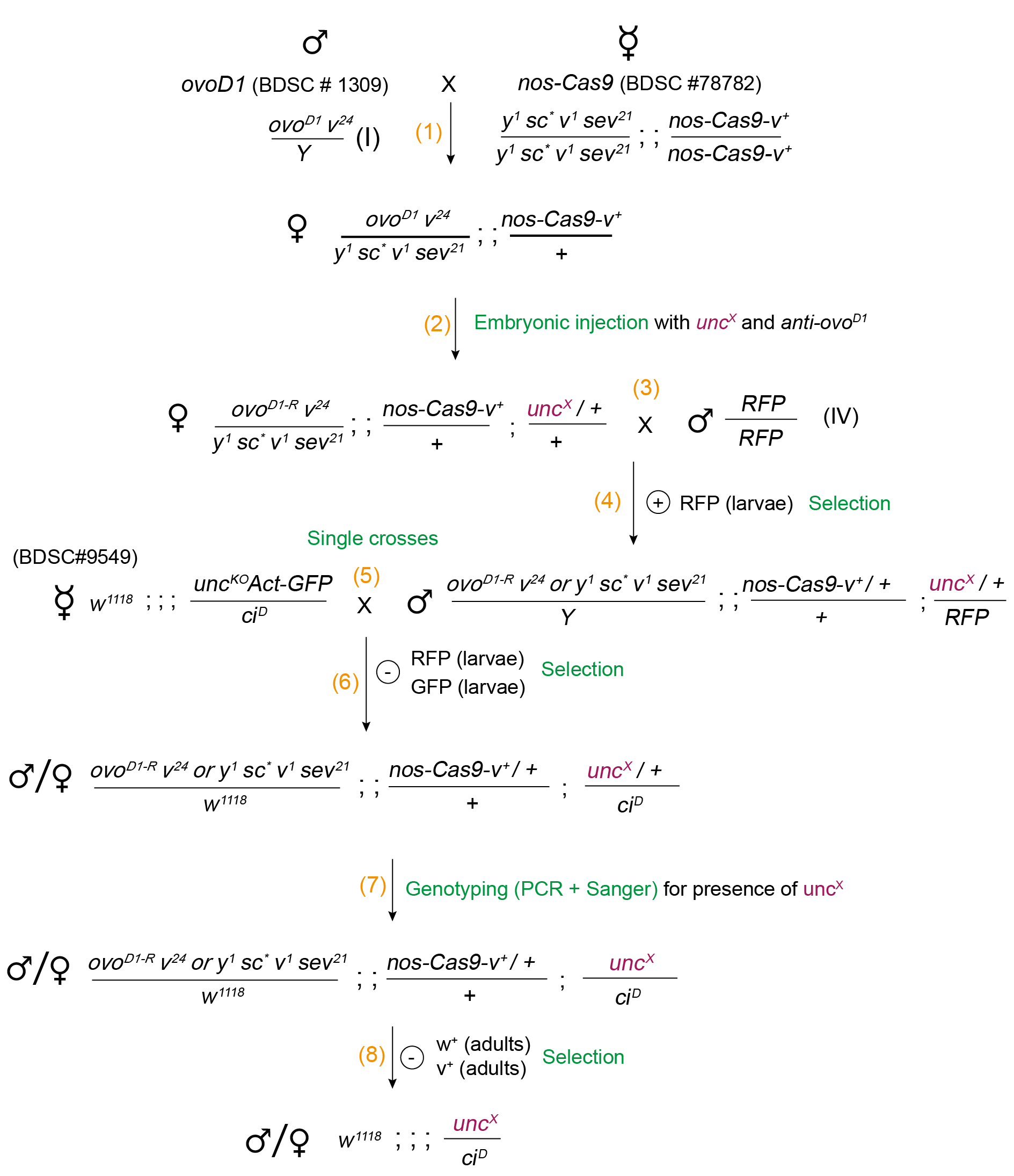


**Figure S3. The crossing scheme used in CRISPR-based mutagenesis.**

(1) ovo^D1^ males were crossed to Cas9-expressing virgins. (2) The offspring embryos were injected with an HDR donor plasmid as well as sgRNA plasmids to introduce the mutation unc^x^ (purple) and additionally an sgRNA plasmid to target the dominant negative ovo^D1^ locus and make female offspring fertile. When one CRISPR/Cas9-editing event happens, e.g. targeting of ovo^D1^, the probability of another editing event is very high. Therefore, fertile females had almost always unc^x^ integrated. (3) The resulting females were crossed to RFR/RFP (IV) flies and (4) selected for RFP positive offspring larvae. (5) The resulting males were single crossed to Ci^D^ carrying virgins to bring the potential unc^x^ mutation over the desired marker Ci^D^ and (6) selected for absence of RFP and GFP. Since the fourth chromosome does not recombine (Hartmann & Sekelsky 2017), no balancer was necessary. (7) The resulting flies were genotyped for the presence of unc^x^. Vials with flies, which did not carry unc^x^ were discarded. (8) Virgins and males were selected for the absence of the white (red eyes) and vermilion (red eyes) markers. For the ethanol resistance assay, the genotypes were back-crossed into the Roman lab Canton S strain background.

| Primer | *5’-3’* sequence | |
| --- | --- | --- |
| To amplify the genomic locus in order to create HDR plasmids | | |
| am_226F | AGTCCCGCGGGGCCATACACGTCCATGTTG | *unc13^#Ctrl 1^*, *unc13^#2,3,4^* |
| am_223R | AGTCCCTAGGGCCTTGTCATTTTAACGCAC |  |
| am_227F | AGTCCCGCGGTGGTAGCCATCATAACACC | *unc13^#Ctrl 2^*, *unc13^#5,6,7^* |
| am_225R | AGTCCCTAGGCGGAAAAGCATCAACCTTTC |  |
| To check for the presence of mutations *unc13^X^* after mutagenesis. | | |
| am_255F | CGTCAACCACAACGCCATTG | *unc13^#2^* |
| am_256R | GGAATTTAAATTGTCAGCGTTGG |  |
| am_245F | GCCAGGGACGGAATCAATAGTG | *unc13^#3^* |
| am_257R | GCCGAATATAAATTTTCCTCGCC |  |
| am_258F | GTGGGAGAAATTAGTTCGACC | *unc13^#4^* |
| am_259R | GATAGAGACTCTACATCGGGC |  |
| am_242F | CAAAAAAGCTCTACAAGCCC | *unc13^#5^* |
| am_252R | GGCCGACCACTTGCTTGTAC |  |
| am_253F | GGCTATTATGTTTTAACGCCG | *unc13^#6^* |
| am_219R | GGATATAGGATATATCAGGGC |  |
| am_254F | GCGGAAGACAAGGCTAACTC | *unc13^#7^* |
| am_252R | GGCCGACCACTTGCTTGTAC |  |
| To check for unwanted mutations at CRISPR sites I-IV | | |
| kg_014F | ACATGTCACTTACCTGAAATC | *CRISPR site I* |
| kg_015R | TTGTGGTTGACGTACTGG |  |
| kg_016F | AACATACTCCCGATTGGCC | *CRISPR sites II* |
| kg_017R | TGACGGTGTCGTTTGCTGA |  |
| kg_018F | GGCTATTATGTTTTAACGCCGAGA | *CRISPR sites III* |
| kg_019R | GTGCCAACGCTATAAAATAACAGG |  |
| kg_020F | GGGTAAATAAGTGTGCGCACC | *CRISPR sites IV* |
| kg_021R | CGTTTACGAAAACTACAGTAAGGTGGTT |  |
| Annealing primers to create sgRNAs | | |
| am­_316F | CTTCGCTGATCCGGGCCCGCTATA | *CRISPR site I* |
| am­_317R | AAACTATAGCGGGCCCGGATCAGC |  |
| am_324F | CTTCGTCATCATCATATACCTCCGT | *CRISPR site II* |
| am_325R | AAACACGGAGGTATATGATGATGAC |  |
| am_326F | CTTCGTTGTCTAAAGGGACGTAACG | *CRISPR site III* |
| am_327R | AAACCGTTACGTCCCTTTAGACAAC |  |
| am_328F | CTTCGCAGTTGAGGGAACATCTAAG | *CRISPR site IV* |
| am_329R | AAACCTTAGATGTTCCCTCAACTGC |  |

**Table S1. Primers used in this study.**

| *CRISPR site* | sgRNA plasmid ID |
| --- | --- |
| *I* | pAM71 |
| *II* | pAM78 |
| *III* | pAM80 |
| *IV* | pAM79 |

**Table S2. sgRNA plasmids used in this study.** sgRNA plasmids are shown, corresponding annealing primers used to create the sgRNA plasmids are listed in Table S1.

| *Encoded mutation* | HDR plasmid ID |
| --- | --- |
| *unc-13^#2-fixed^* | pTL957 |
| *unc-13^#3-fixed^* | pTL958 |
| *unc-13^#4-fixed^* | pTL963 |
| *unc-13^#5-fixed^* | pTL965 |
| *unc-13^#6-fixed^* | pTL966 |
| *unc-13^#7-fixed^* | pTL967 |
| *unc-13^Ctrl1-fixed^* | pTL962 |
| *unc-13^Ctrl2-fixed^* | pTL964 |

**Table S3. Homology-directed repair plasmids used in this study.** The generation of the HDR plasmids was previously described (Götze et al., 2022). Modifications of the sgRNA binding sites and PAMs were performed by GenScript (USA).

| Patient | Variant/  Mutation | *Genotype* | Fly strain number | Mutagenesis  identifier of fly strain | Internal ID of flies crossed over *ci^D^* | Internal ID of flies crossed over *Gat^eya^* |
| --- | --- | --- | --- | --- | --- | --- |
| 2 | #2 | *unc13^#2^ = unc13C^C69F^* | 1  2  3  4  5 | 1A#52^$^*^&^  1B#29  1D#64  1D#65  1E#19 | DL129  DL131  DL132  DL130  DL133 | DL151  DL152  DL153  DL154  DL155 |
|  | #3 | *unc13^#3^ = unc13C^A319E^* | 1  2  3  4  5 | 2A#32^$^*^&^  2B#72  2C#4  2C#24  2C#25 | DL119  DL123  DL121  DL120 DL122 | DL160  DL157  DL156  DL161  DL162 |
| 3 | #4 | *unc13^#4^ = unc13C^R548C^* | 1  2  3 | 1A#1^$^*^&^  1B#1  1C#7* | DL140  DL141  DL142 | DL166  DL174  DL173 |
|  | #5 | *unc13^#5^ = unc13C^T1104M^* | 1  2  3 | #151^$^*^&^  #153  #154 | DL124  DL126  DL125 | DL163  DL158  DL159 |
| *4* | #6 | *unc13^#6^ = unc13C^T1053I^* | 1  2  3 | 2A#1  2A#6  2C#5*^&$^ | DL144  DL145  DL143 | DL169  DL170  DL171 |
|  | #7 | *unc13^#7^ = unc13C^I1189T^* | 1  2  3 | 3A#3  3B#4*^&$^  3A#8 | DL147  DL148  DL146 | DL164  DL165  DL172 |
| Wildtype *Ctrl1* | | *unc13^Ctrl 1^* | n.a. | 1C#52*^&^ | DL135 | DL167 |
| Wildtype *Ctrl2* | | *unc13^Ctrl 2^* | n.a. | 3D#128*^&^ | DL127 | DL168 |

**Table S4. Fly strains generated for this study.** Fly strains with Drosophila unc13 mutations mimicking patients’ UNC13C variants. Fly strains used for Mendelian experiments, where the patient phenotype unc13^X^/unc13^Y^ was recreated (Figure 3), are marked with $. Fly strains used for negative geotaxis (Figure 4A,B) and lifespan experiments (Figure 4C) are marked with *. Fly strains used for ethanol sensitivity experiments are marked with &. The strains utilised in experiments were selected based on the availability of a high number of virgin females and males. Related to Figure 1 of the main text.

| Short genotype | Chr. | Genotype | Usage | Internal ID | External ID |
| --- | --- | --- | --- | --- | --- |
| *ovo^D1^* | X | *ovo^D1^ v^24^/C(1)DX, y^1^ w^1^ f^1^* | CRISPR-Cas9 targeting | n.a. | BDSC #1309 |
| *nanos-Cas9* | X, 3 | *y^1^ sc^*^ v^1^ sev^21^; ; P{y^+t7.7^ v^+t1.8^ =nanos-Cas9.R}attP2* |  | n.a. | BDSC #78782 |
| *vas-Cas9* | X, 2 | *w^1118^; PBac{y^mDint2^GFP^E.3xP3^=vas-Cas9} ^VK00037^/CyO, P{w+^mC^ = Tb^1^}Cpr^CyO-A^* |  | n.a. | BDSC #56552 |
| *vas-Cas9* | X | *y^1^ M{GFP^E.3xP3^=vas-Cas9.RFP-}ZH-2A w1118* |  | n.a. | BDSC #55821 |
| *RFP/RFP* | X, 4 | *w^*^; M{3xP3-RFP.attP}ZH-102D* | Crosses to create *unc13^X^* genotypes | DL0177 | Made from  BDSC #24488 |
| *unc^KO^/ci^D^* | X, 4 | *w^1118^; ; ; P{w^+mC^=ActGFP}unc-13^GJ^/In(4)ci^D^, ci^D^ pan^ciD^* | Crosses to create *unc13^X^* genotypes. Mendelian experiments | DL0093 | BDSC #9549 |
| *unc^KO^/pan^2^* | X, 4 | *w^1118^; ; ; P{w^+mC^=ActGFP}unc-13^GJ^/pan^2^* |  | DL0094 | BDSC #4759 |
| *w1118* | X | *w1118* | Mendelian experiments | *w1118* | n.a. |

**Table S5. Fly strains used in this study.**

Genotypes of fly strains used in this study. Chr., chromosome

|  | *Ctrl 1/*  *KO* | *Ctrl 2/*  *KO* | *#2/*  *KO* | *#3/*  *KO* | *#2/*  *#3* | *#4/*  *KO* | *#5/*  *KO* | *#4*  */#5* | *#6/*  *KO* | *#7/*  *KO* | *#6/*  *#7* | *Pos.*  *Ctrl* |
| --- | --- | --- | --- | --- | --- | --- | --- | --- | --- | --- | --- | --- |
| Mean | 9,85 | 9,95 | 8,95 | 9,85 | 9,50 | 9,85 | 9,50 | 9,85 | 9,90 | 9,95 | 9,85 | 5,05 |
| SD | 0,37 | 0,22 | 1,10 | 0,37 | 0,61 | 0,37 | 0,76 | 0,37 | 0,31 | 0,22 | 0,37 | 1,19 |
| SEM | 0,08 | 0,05 | 0,25 | 0,08 | 0,14 | 0,08 | 0,17 | 0,08 | 0,07 | 0,05 | 0,08 | 0,27 |

**Table S6. Negative geotaxis.**

Results of negative geotaxis experiments show the mean number of flies out of ten (including SD, SEM), which climbed higher than 8 cm after 10 seconds in 20 runs (n=20). Genotypes are abbreviated as follows: Ctrl 1/KO stands for unc13^Ctrl 1^/unc13^KO^, #2/KO means unc13^#2^/unc13^KO^, etc. Related to Figure 4B and Table S7.

|  | *Ctrl 1/*  *KO* | *Ctrl 2/*  *KO* | *Pos.*  *Ctrl* | *#2/*  *KO* | *#3/*  *KO* | *#2/#3* | *#4/*  *KO* | *#5/*  *KO* | *#4/#5* | *#6/*  *KO* | *#7/*  *KO* | *#6/#7* |
| --- | --- | --- | --- | --- | --- | --- | --- | --- | --- | --- | --- | --- |
| *Ctrl 1/*  *KO* | X | 1 | <0.001 | 1 | 1 | 1 | 1 |  | 1 |  |  |  |
| *Ctrl 2/*  *KO* |  | X | <0.001 |  |  |  |  | 1 | 1 | 1 | 1 | 1 |
| *Pos.*  *Ctrl* |  |  | X | <0.052 | <0.001 | 0.002 | <0.001 | <0.001 | <0.001 | <0.001 | <0.001 | <0.001 |

**Table S7. Negative geotaxis statistical comparisons.**

Statistical comparison of results from negative geotaxis experiment in Table S6. Since the data were non-normally distributed (Shapiro-Wilk test), multiple comparison analyses (groups compared to every other group) were done using the Kruskal-Wallis test (=One-way ANOVA on ranks) followed by Dunn’s test for multiple comparisons. While every group was compared to every other group, we show the relevant comparisons only, i.e. the comparison against the positive control (Pos. Ctrl) and against the relevant negative control genotypes, i.e., unc13^Ctrl 1^/unc13^KO^ or unc13^Ctrl 2^/unc13^KO^. Genotypes are abbreviated: Ctrl 1/KO stands for unc13^Ctrl 1^/unc13^KO^, #2/KO stands for unc13^#2^/unc13^KO^, etc. Related to Figure 4B, Table S6.

|  | *#2* | *#3* | *#4* | *Ctrl1* | *#5* | *#6* | *#7* | *Ctrl 2* |
| --- | --- | --- | --- | --- | --- | --- | --- | --- |
| N | 18 | 18 | 16 | 18 | 32 | 29 | 29 | 20 |
| Minimum | 12.78 | 11.67 | 11.43 | 11.43 | 9.231 | 13.57 | 13.75 | 12.50 |
| 25% percentile | 16.12 | 16.95 | 12.86 | 12.20 | 13.24 | 14.77 | 16.77 | 16.44 |
| Median | 17.75 | 19.38 | 13.61 | 12.76 | 14.23 | 16.67 | 18.13 | 18.06 |
| 75% percentile | 23.19 | 22.06 | 15.94 | 13.75 | 16.59 | 18.83 | 19.08 | 20.00 |
| Maximum | 25.00 | 25.00 | 17.00 | 17.86 | 25.00 | 21.50 | 23.57 | 21.88 |

**Table S8. Ethanol sensitivity.**

Descriptive statistical analyses of LoRR (in minutes) are shown. N, number of runs. Genotypes are abbreviated: Ctrl 1 stands for homozygous unc13Ctrl 1, #2 stands for homozygous unc13#2, etc. Related to Figure 5B.

| Patient | Human  variant | Exchange in human | Exchange in *Drosophila* |
| --- | --- | --- | --- |
| *2* | #2 *unc13C^C69F^* | Cys (special) → Phe (hydropobic)  active exchange | Val (special) → Phe (hydropobic)  inert exchange |
|  | #3 *unc13C^A319E^* | Ala (hydrophobic) → Glu (negative)  active exchange | Asp (negative) → Glu (negative)  inert exchange |
| *3* | #4 *unc13C^R548C^* | Arg (polar) → Cys (special)  active exchange | Asp (negative) → Cys (special)  active exchange |
|  | #5 *unc13C^T1104M^* | Thr (polar) → Met (hydrophobic)  active exchange | conserved |
| *4* | #6 *unc13C^T1053I^* | Thr (polar) → Ile (hydrophobic)  active exchange | Ala (hydrophobic) → Ile (hydrophobic)  active exchange |
|  | #7 *unc13C^I1189T^* | Ile (hydrophobic) → Thr (polar)  active exchange | conserved |

**Table S9 Putatively pathogenic *UNC13C* variants and corresponding *Drosophila* mutations.**

An overview of patient-derived *UNC13C* variants, their resulting amino acid changes in humans, and the engineered substitutions in the Drosophila Unc13 protein. Amino acids were categorised according to their side chains into hydrophobic, negative, polar. Cysteines were marked as special, because of their ability to build disulfide bridges. Amino acid substitutions are categorised as "active" (non-conservative), when a substitution resulted in an amino acid of the same category or "inert" (conservative), when a substitution resulted in an amino acid from a different category.

References

Hartmann MA & Sekelsky J (2017). The absence of crossovers on chromosome 4 in Drosophila melanogaster: Imperfection or interesting exception?, *Fly* **11**, 253–259; DOI: 10.1080/19336934.2017.1321181.
